# Advanced Deep Learning and Machine Learning Techniques for MRI Brain Tumor Analysis: A Review

**DOI:** 10.3390/s25092746

**Published:** 2025-04-26

**Authors:** Rim Missaoui, Wided Hechkel, Wajdi Saadaoui, Abdelhamid Helali, Marco Leo

**Affiliations:** 1 Laboratory of Micro-Optoelectronics and Nanostructures (LMON), University of Monastir, Avenue of the Environment, Monastir 5019, Tunisia; missaouirima22@gmail.com (R.M.); hechkelwided@gmail.com (W.H.); abdelhamid.helali@gmail.com (A.H.); 2 National High School of Engineering of Tunis (ENSIT), 5 Rue Taha Hussein–Montfleury, Tunis 1008, Tunisia; 3 LRMAN Laboratory, Higher Institute of Applied Sciences and Technology of Kasserine (ISSAT), Kasserine 1200, Tunisia; wajdi.enit@gmail.com; 4 Institute of Applied Sciences and Intelligent Systems, National Research Council of Italy, 73100 Lecce, Italy

**Keywords:** brain tumors, machine learning (ML), deep learning (DL), magnetic resonance imaging (MRI)

## Abstract

A brain tumor is the result of abnormal growth of cells in the central nervous system (CNS), widely considered as a complex and diverse clinical entity that is difficult to diagnose and cure. In this study, we focus on current advances in medical imaging, particularly magnetic resonance imaging (MRI), and how machine learning (ML) and deep learning (DL) algorithms might be combined with clinical assessments to improve brain tumor diagnosis. Due to its superior contrast resolution and safety compared to other imaging methods, MRI is highlighted as the preferred imaging modality for brain tumors. The challenges related to brain tumor analysis in different processes including detection, segmentation, classification, and survival prediction are addressed along with how ML/DL approaches significantly improve these steps. We systematically analyzed 107 studies (2018–2024) employing ML, DL, and hybrid models across publicly available datasets such as BraTS, TCIA, and Figshare. In the light of recent developments in brain tumor analysis, many algorithms have been proposed to accurately obtain ontological characteristics of tumors, enhancing diagnostic precision and personalized therapeutic strategies.

## 1. Introduction

The brain is the most complex organ in the human body and plays an important part in daily living [1]. The majority of bodily functions are handled by it, including decision making, organizing, integrating, analyzing, and sending signals to the other body parts. The anatomical architecture of the human brain is extremely complex [2]. As an aberrant development of tissues, tumors are considered the second most common cause of mortality. They originate from abnormal cells that proliferate uncontrollably and fail to undergo the normal cell life cycle of growth and apoptosis. Tumors are often recognized by the organ or bodily component from which they started. Thus, tumors that arise from any area of the brain or skull, including the brain’s nerves, tissues, membranes, or bones, are classified as brain tumors, which have a 70% death rate [3]. According to Cancer.Net, about 251,329 individuals worldwide lost their lives to malignant brain and central nervous system (CNS) tumors in 2020, while approximately 308,102 people had a primary brain or spinal cord tumor diagnosis. The primary or metastatic origin of brain tumors determines their classification. Secondary or metastatic brain tumors originate from malignant elements in other regions of the body that disseminate to the brain. In contrast, primary brain tumors are constituted by the cells of the brain tissue itself [4]. Each year, large numbers of people are diagnosed with brain tumors, approximately 250,000, and 2% of these cases are confirmed as malignant [5].

Due to their complexity and variety, brain tumors present substantial obstacles in clinical practice. A diagnosis of a brain tumor, along with the subsequent segmentation and classification of the tumor, are of critical importance to the effective planning of treatment and the care of the patient. Technologies employed in the study of brain activity and architecture include positron emission tomography (PET), computed tomography (CT), single photon emission computed tomography (SPECT), and magnetic resonance imaging (MRI). Because of its superior contrast resolution and anatomic detail without ionizing radiation, magnetic resonance imaging (MRI) is a favored modality among the several imaging techniques used to confirm brain tumors [6]. When a brain is subjected to magnetic resonance imaging (MRI), multiple three-dimensional image data are produced. These images include the coronal, axial, and sagittal views of the brain at various depths. Inter-slice gap, slice thickness, and image quality also differ according to sampling techniques used for obtaining spectral data with respect to magnetic field strength [7]. An MRI image can also be different depending on the sequence of pictures. This can be achieved with nuclear magnetic resonance (NMR) signals of many radio frequencies that have a range in the time between signal pulses and echoes, thereby generating diverse sequences (field-attenuated inversion recovery (FLAIR), T1-weighted, T2-weighted, etc.), featuring valuable parameters. Different types of image sequences highlight specific brain or skull areas and can be utilized to study different regions in the human brain.

The analysis of brain tumors is a crucial area of medical imaging and involves several related processes, including detection, segmentation, classification, and prediction. The detection stage uses sophisticated algorithms for identifying the probable tumor regions in MRI images. The next step is segmentation, which determines exactly where the observed tumors are located and their size. Classification further details the diagnosis into type and grade, which are important for making treatment decisions. Finally, predicting how the tumor will behave and respond to treatment contributes to personalized therapy. These steps serve to make brain tumor diagnosis and treatment more exact and successful, which is best for patients.

Radiologists usually perform brain tumor analysis from MRI sequences manually. However, because of human fatigue, the high number of MRI slices per patient, inter-observer variability, and the growing patient population, this manual procedure is highly demanding, time-consuming, and error-prone [8]. This is generally unreliable because manual delineation can be subject to variation, which in turn can affect radiomic analysis, and therefore, lead to inconsistent results. Tumor type significantly impacts the performance of deep learning models, particularly in terms of segmentation accuracy. Solid tumors, with well-defined boundaries, typically result in higher accuracy, as the clear delineation allows for more straightforward detection. However, glioblastomas, characterized by their diffuse and infiltrative nature, present a more challenging task for segmentation, often leading to lower accuracy. The irregular boundaries of glioblastomas make it difficult to clearly define the tumor region, which can negatively affect segmentation and overall performance [9]. Such cases, in particular poor-contrast or artifact-affected MRI scans, are difficult for humans to detect, and thus, provide room for improvement by deep learning models. These models can learn subtle features and contextual patterns that may not be visible to the human experts. However, their performance depends heavily on the quality and diversity of training data. Image artifacts, such as noise or motion blur, can degrade accuracy, but this damage can be countered with preprocessing, attention-based architectures, and artifact-robust training techniques [10].

Due to the rapid expansion of global data collecting, recent advances in deep learning (DL) techniques have drastically altered medical image processing, particularly for brain tumor identification and classification from an MRI scan. Previously, the assessment of tumors was based on qualitative parameters like tumor density, cellular resolution within the lesion, and anatomic continuity with other tissues. Yet, this is now possible due to technological advances because neuropathology can be assessed quantitatively and the size, form, and textural patterns of tumors can be determined. The significant progress in brain tumor analysis can be credited to the breakthroughs in artificial intelligence (AI) and computer vision (CV) [11].

Researchers are increasingly using artificial intelligence (AI) technologies, particularly machine learning (ML) and deep learning (DL), to enhance or automate diagnostic procedures in order to overcome challenges associated with manual brain tumor diagnosis. Traditional AI approaches, such as rule-based expert systems, are limited by their inability to generalize across the complex, heterogeneous nature of brain tumors. In contrast, machine learning (ML) methods like support vector machines (SVMs), random forest (RF), and k-nearest neighbors (k-NN) may categorize tumors using characteristics that are generated from radiomic signatures or MRI data. However, these ML models depend heavily on handcrafted feature extraction, which can limit adaptability to new cases or modalities.

Deep learning, particularly convolutional neural networks (CNNs), has revolutionized brain tumor analysis by learning hierarchical features directly from raw imaging data. The implementation of CNNs excludes manual features from the analysis process since they work remarkably well for complex procedures like tumor segmentation detection and classification. Recent models also incorporate attention mechanisms and transformer-based architectures, further improving performance on tasks requiring spatial context and feature interaction. DL models can process large-scale MRI datasets and deliver reliable, automated outputs that support clinical decision making and reduce inter-observer variability.

The accessibility of implementing ML and DL models into clinical implementation has increased. Medical experts and researchers can begin using these technologies through open-source tools such as MIPAV (Medical Image Processing, Analysis, and Visualization) [12] as well as other open-source tools for image-based analysis. The combination of free cloud platforms like Google Colab enables developers to work on DL models through TensorFlow (v2.12) alongside Keras (v2.12) and PyTorch (v2.0.1) libraries. These resources, along with online courses and tutorials, lower the entry barrier and facilitate the adoption of AI in medical imaging workflows.

In this review paper, we are going to discuss many aspects of MRI images and try to understand how these types work in the study process. It also discusses the different facts about brain tumors to improve knowledge. Throughout this paper, we offer a thorough overview of current deep learning, machine learning, and hybrid models used in the analysis of brain tumors for four primary applications including detection, segmentation, and classification as well as survival prediction with details about methods employed in datasets used to evaluate these networks and performance metrics. The article ends with a short recap of the findings and suggestions for future research.

## 2. Overview

### 2.1. Magnetic Resonance Imaging (MRI)

**Figure 1:**
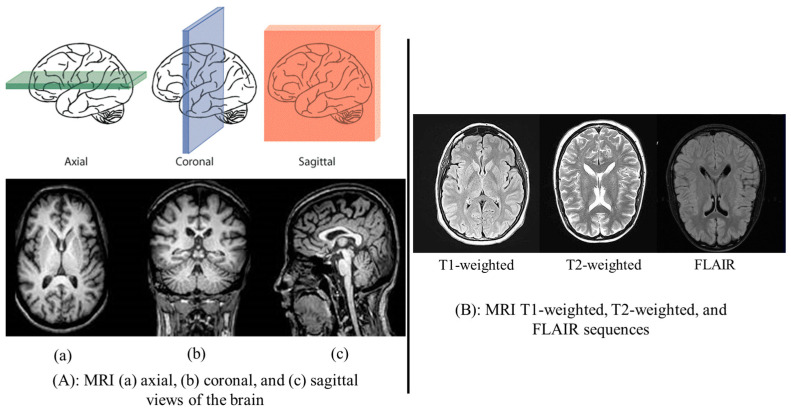
MRI scans of planes and sequences.

Magnetic resonance imaging (MRI) is a well-established and widely utilized clinical diagnostic technique. Its adaptability enables better viewing of an organ’s structural features. It also allows for multi-modal imaging, provides cross-sectional views with consistent resolution across all slices, and facilitates thorough diagnostic examinations [13]. MRI is highly beneficial for detecting brain tumors and provides greater accuracy than other non-invasive techniques because it has remarkable contrast that supplies accurate information on the soft tissue inside the brain. MRI is a type of imaging that allows for full viewing of the brain or spinal cord (vasculature) by taking pictures in three separate planes: coronal, axial, and sagittal. MRI may be used to image many different body sections, since it is excellent at seeing soft tissues. MRI is invaluable for imaging various parts of the body, including detecting tumors and aneurysms [14] and distinguishing between white matter and gray matter in the brain. MRI can also be used on the spinal cord [15], neurons [16], muscles [17], and ligaments [18]. MRI is also a safe way to take images compared to computed tomography (CT), since ionizing radiation can be avoided by using its magnetic fields and RF pulses. MRI produces 2D or 3D high-contrast imaging. Figure 1A indicates the primary MRI planes that provide an in-depth view of brain anatomy.

Furthermore, MRI images can be different in each case, which depends on the use of image sequences with specific radio frequencies, reaction times, and echo times of pulse signals, which provide various types of images. The three most often used magnetic resonance imaging sequences for brain analysis are T1-weighted, T2-weighted, and fluid-attenuated inversion recovery (FLAIR) [19], each of them capturing distinct characteristics required for full brain analysis. Gray and white contrast can be achieved using T1-weighted scanning. T2-weighted is suited for diseases that cause water accumulation in brain tissue due to its water content sensitivity. T1 and T2 weighted images are used to identify CSF (cerebrospinal fluid), which is a colorless fluid present in the brain and spinal cord that looks dark in T1 and T2 weighted imaging. The FLAIR scanning, which is comparable to T2-weighted imaging, is used to distinguish MR imaging abnormalities in the brain from CSF. CSF edema can be localized using the FLAIR scan. Figure 1B displays the T1-weighted, T2-weighted, and FLAIR scans, and Table 1 displays their echo times (the interval between the receiving of radio frequency remittance echo signals) and repetition times (the interval between two successive pulse sequences).

In addition to conventional sequences such as T1, T2, and FLAIR, advanced diffusion-based MRI methods, such as Diffusion-Weighted Imaging (DWI) and Diffusion Tensor Imaging (DTI), provide crucial microstructural insights by monitoring the movement of water molecules in tissue. By modeling diffusion as a three-dimensional tensor, DTI, an extension of DWI, makes it possible to quantify white matter integrity using measures like mean diffusivity (MD) and fractional anisotropy (FA). As they help with tumor grading, identifying gliomas from metastases, and tracking therapy response (e.g., separating actual progression from pseudo progression), these approaches are extremely useful in the field of neuro-oncology [20]. However, compared to T1, T2, and FLAIR-based sequences, DWI/DTI generally suffer from lower spatial resolution and increased motion sensitivity. Emerging techniques like Diffusion Kurtosis Imaging (DKI) and Neurite Orientation Dispersion and Density Imaging (NODDI) further enhance specificity by modeling non-Gaussian diffusion or estimating neurite density. Despite their clinical potential, these advanced models require longer acquisition times and remain less common in routine practice.

### 2.2. Brain Tumor Analysis Using Deep Learning and Machine Learning

Currently, machine learning (ML), which is often employed in the medical imaging field, has provided powerful tools for the effective interpretation of intricate medical data. The goal of ML, which is defined as a system’s capacity to learn from training data and apply this knowledge in future problems without human intervention, plays a vital role in brain tumor analysis using MRI. By performing features and trends, the ML system helps in screening brains using MRI, as well as in predicting and treating brain tumors. The main goal of certain types of ML algorithms is to generalize the learned knowledge and make a correct prognosis on new data, adjusting the algorithm and improving its prognosis in the learning data.

**Figure 2:**
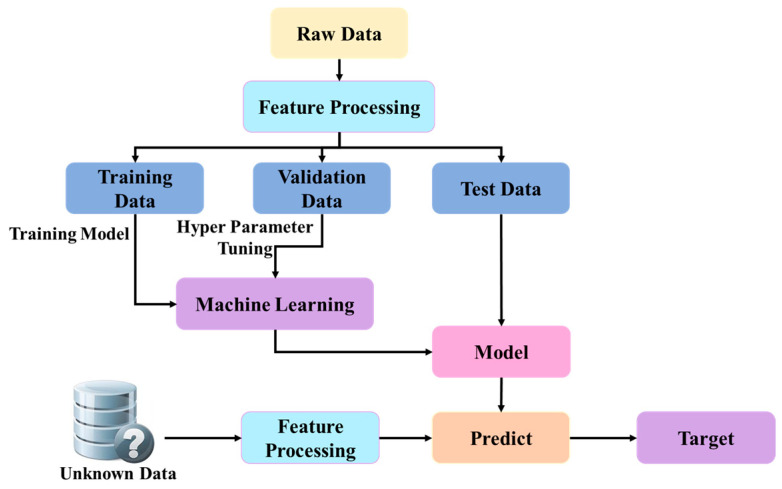
Machine learning process.

Support vector machines (SVMs), feed-forward neural networks, artificial neural networks (ANNs) [21], backpropagation neural networks (BPNNs) [22], and k-nearest neighbors (KNN) are among the fundamental ML learning algorithms that have found extensive use in medical imaging. These algorithms can enhance, for instance, analysis of MRI scans and can help improve diagnosis of brain tumors. Figure 2 epitomizes the basic structure of an ML model, which acquires its knowledge of new situations from data and undergoes assessment and optimization to enable it to make correct predictions regarding unseen data. However, there are several issues which exist with current ML approaches, including the requirement of large sets of annotated data and variability in imaging protocols, which may affect the interpretability of results.

In recent years, deep learning (DL), which is under the broad umbrella of machine learning (ML), has been developed to be a more advanced technique than traditional ML. Unlike the traditional ML algorithms that depend on the features being extended, DL models learn feature hierarchies from the raw data, which allow them to outperform standard ML models for several tasks. Convolutional neural networks (CNNs) are becoming increasingly popular for various high-demanding medical image analysis tasks due to their capacity to autonomously process massive volumes of images and distinguish complex patterns such as forms, edges, and textures.

**Figure 3:**
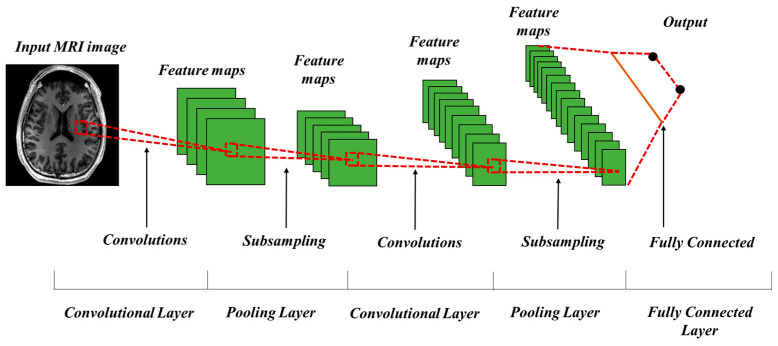
General CNN architecture.

The CNN architecture, as represented in the Figure 3, consists of a series of different layers, each performing their own mathematical operation on the input data to extract features starting from very general to very specific features. Convolutional is the core operation a CNN, responsible for detecting patterns and features in the input data. Each filter generates a feature map, representing various aspects of the input image. Mathematically, the convolution operation is defined as:

(1) $$F_{\left(i,\;\;\;j\right)}={\left(G\times H\right)}_{\left(i,\;\;\;j\right)}=\sum_{m}\sum_{n}G_{\left(m,\;\;\;n\right)}H_{\left(i-m,\;\;\;j-n\right)}$$

where $F_{\left(i,j\right)}$ is the output feature map at position (*i*, *j*), $G_{\left(m,\;n\right)}$ is the input image, and $H_{\left(i-m,\;j-n\right)}$ represents the filter applied to the input. Feature map spatial dimensions reduction during pooling decreases computational cost and increases the network’s resilience to geographic fluctuations in the input. Max pooling stands as the widely employed pooling operation that selects the largest value from feature map areas represented by:

(2) $$P_{ij}=\max X_{ab},\;a,\;b\;\epsilon N_{ij}$$

where $P_{ij}$ is the output at position (*i*, *j*), $X_{ab}$ represents the elements in the pooling window, and $N_{ij}$ is the neighborhood covered by the pooling kernel. Finally, features learned by previous layers are integrated by the fully connected layer for the final prediction or classification. Each neuron in this layer connects to all activations from the preceding layer to build a dense structure that discovers advanced connections among features. The output of an FC layer is computed as:

(3) $$y=\sigma \left(W_{X}+b\right)$$

where *X* is the input vector, *W* is the weight matrix, *b* is the bias vector, and σ represents the activation function.

**Figure 4:**
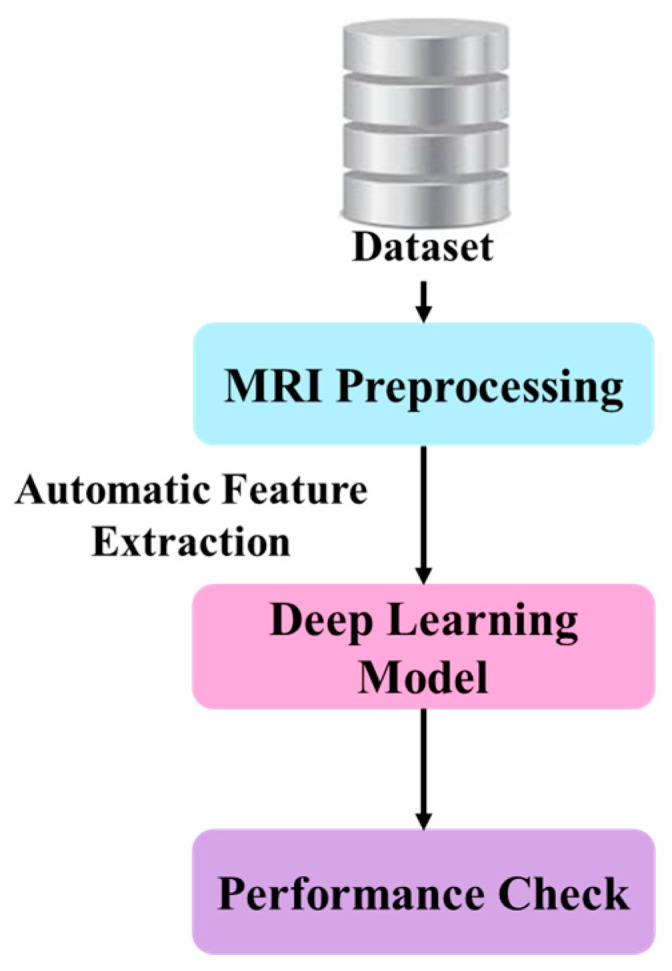
Deep learning process [23].

In MRI-based brain tumor analysis, DL models have inevitably reformulated the default approaches by eliminating the manual process of feature extraction, thus improving the quality of the analysis. The workflow normally starts with the acquisition and initial processing of MRI scans for analyzing the data. The previously processed images are passed to a DL model, like CNN, to automatically extract features without manual intervention. Then, as illustrated in Figure 4, the model’s performance is assessed to assure its accuracy and reliability in brain tumor analysis utilizing MRI.

Moving from ML to DL has made brain tumor analysis much more accurate and reliable, lowering the manual effort on diagnostics and helping achieve better diagnostic results. Despite this, challenges such as the requirement for extensive training datasets and simplistic model interpretability continue to remain, which applies to both DL and ML methods. The utilization of DL may represent the future in brain tumor analysis and holds powerful potential to improve diagnosis and patient outcomes with personalized treatment strategies.

### 2.3. Performance Evaluations

The performance metrics used for brain tumor analysis include accuracy, specificity, loss, recall, precision, and F1 score. These metrics are widely employed in disease diagnosis for various models and methods performance evaluation. For example, according to the confusion matrix, brain tumors can be categorized as true positive (TP, original class: tumor, predicted class: tumor), false positive (FP, original class: non-tumor, predicted class: tumor), false negative (FN, original class: tumor, predicted class: non-tumor), or true negative (TN, original class: non-tumor, predicted class: non-tumor). The most widely used metrics are described below [24].

- Accuracy (*ACC*): The model’s accuracy can be expressed as the proportion of correctly classified data.

(4) $$ACC=\frac{TP+TN}{TP+TN+FP+FN}$$

- Precision (*P*): The ratio of accurately predicted positive observations to the total number of expected positive observations.

(5) $$P=\frac{TP}{TP+FP}\;$$

- Recall (*R*): Recall is the proportion of relevant results that the algorithm correctly identifies.

(6) $$R=\frac{TP}{TP+FN}$$

- Specificity *S_p_*: This calculates the percentage of real negative occurrences that the algorithm accurately classifies as negative.

(7) $$S_{p}=\frac{TN}{TN+FP}\;$$

- *F*1-measure: This is calculated by taking the harmonic mean of recall and accuracy. A score of 1 indicates the highest level of accuracy and recall, which is considered to be the optimal result.

(8) $$F1-measure=2\times \frac{Precision\times Recall}{Precision+Recall}$$

## 3. Brain Tumor Analysis Techniques

**Figure 5:**
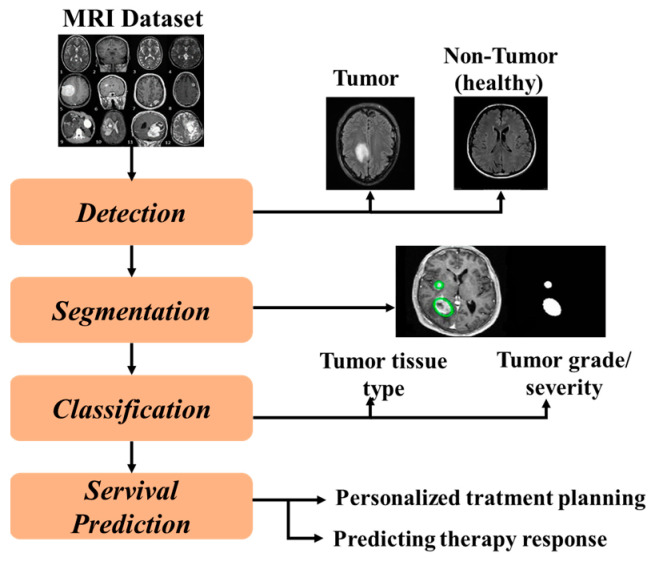
Brain tumor analysis tasks.

As illustrated in Figure 5, brain tumor analysis tasks may be classified into four principal categories: detection, segmentation, classification, and prediction of treatment outcome and prognosis. Researchers solve these issues using a variety of deep learning (DL), machine learning (ML), and hybrid approaches on different types of brain imagery, essentially MRI scans. These systems, based on AI, enable the analysis to be accurate and quicker, thereby helping in earlier diagnosis, accurate localization, classification, and prediction in a more precise way.

The tumor detection task consists of a binary decision for each medical image by identifying the absence or presence of a tumor to be detected so as to not only make an early diagnosis but also timely intervention. The task of tumor segmentation requires us to locate and extract an accurate region in all cells above a certain threshold that contains a cell so far as treatment preparation is concerned because it represents the tumor size, location, and shape. The task of tumor classification further classifies the segmented tumor according to relevant omics-based characteristics, such as severity (benign or malignant) and tissue types (e.g., core tumor, edema, enhancing-highlight region). A reliable classification aids in prognosis, helping oncologists to select the right therapeutic regimens. Finally, the prediction task relates to anticipating what will happen based on past data and its impact from medical imagery or clinical data on patient prognosis by evaluating survival rate, and therapeutic response estimation to find possible troubles ahead. This task is critical for personalized treatment planning and enhancing patient management. In the following section, we will explore the diverse methodologies used for each of these tasks, beginning with machine learning techniques and progressing to deep learning techniques, to understand how they contribute to improved efficiency and accuracy in brain tumor analysis, improving patient care and outcomes.

### 3.1. Machine Learning Techniques

Machine learning techniques have been increasingly adopted as central tools in many of the traditional MRI-based brain analysis components, such as detection, the characterization of abnormal brain structures, classification, and survival prediction. Such methods are of significant value due to their capacity to facilitate the learning and extraction of useful features from complex MRI data in an automated manner, which aligns with the unique challenges associated with brain imaging.

#### 3.1.1. Tumor Detection Using Machine Learning

H. Abdallah et al. [25] proposed a neural network that uses feed-forward back-propagation for brain tumor detection task, yielding scores of 99% and 97.9%, respectively, for accuracy and sensitivity when trained on 239 MRI images. A novel approach to the identification of brain tumors was introduced by N. Arunkumar et al. [26], utilizing the K-means and artificial neural networks (ANN) techniques. First, the initial segmentation of the MR images into regions of interest is performed using K-means clustering based on texture features. Then, a trained ANN was utilized to detect the tumor from segmented data. The proposed method achieved performance evaluation scores of 94.07%, 90.09%, and 96.78% on the BraTS 2015 dataset for accuracy, sensitivity, and specificity. J. Amin et al. [27] developed an automated technique by using the MRI data, in which they applied K-means clustering for lesion classification after removing artifacts and using the Brain Surface Extractor (BSE) for brain extraction. Using a hybrid feature set of intensity, texture, and shape, three SVM classifier variants were trained and tested, resulting in an accuracy of 97.1%, an ROC AUC of 98% ROC AUC, a specificity of 98%, and a sensitivity of 91.9%. The described method can significantly enhance clinical practice since it allows precise and early brain tumors detection, minimizes the number of diagnostic errors, and assists radiologists in their decision-making process. The high level of accuracy and repeatability allows using the algorithm in clinical applications, adding efficiency and better patient outcomes. A technique based on the sub-sampled contourlet transform (NSCT) and the adaptive neural fuzzy inference system (ANFIS) was proposed by Selvapandian et al. [28], in which the NSCT enhances abnormal regions, and ANFIS classifies images as normal or glioma. This method achieved high sensitivity (92.3% and 96.2%), specificity (96.2% and 95.1%), and accuracy (95.9% and 96.4%) on BRATS 2015 Leaderboard and Challenge datasets. H. T. Zaw et al. [29] applied Naïve Bayes classification to identify tumor areas in magnetic resonance images. It accurately identifies tumors, including challenging regions like the midbrain, achieving 94% accuracy, with 81.25% success in detecting tumors and no misclassification of non-tumor cases when tested on 50 MRI images. A machine learning (ML) method was introduced by A. Stadlbauer et al. [30] for multi-modal classification of brain tumor regions using advanced and physiological MRI. Testing nine ML algorithms on radiomic features from 167 patients with five tumor types, they created 135 classifiers. Adaptive boosting and random forest models surpassed radiologists with 87.5% accuracy, 86.2% precision, 77.4% F-score, and 88.6% AUROC. The study emphasized ML-based radiophysiomics’ potential but suggested deep learning to reduce preprocessing complexity. A brain tumor detection and segmentation methodology was introduced by Bhimavarapu et al. [31], who made use of morphology and FCM clustering. To categorize the tumors according to physical characteristics like color intensity and texture, they employed an Extreme Learning Machine. Tested on the Figshare dataset and a combined dataset from Figshare, SARTAJ, and Br35H datasets, this method showed scores of 98.56%, 99.14%, and 99.25%, respectively, for accuracy, precision, and recall, outperforming previous approaches with an improvement of 1.21% to 6.23% and highlighting its promise for real-time tumor detection. It can be clinically applied in increasing the efficiency of the detection and classification of brain tumors, aiding in early diagnosis and personalized treatment planning. These machine-learning-based approaches for brain tumor detection are summarized in Table 2. 

Data imbalances within datasets lead to performance variations in models such as support vector machines (SVMs), which achieved 97.1% accuracy [27], and backpropagation neural networks, which reached 99% [25]. The effectiveness of SVMs decreases when handling noisy data, as they rely on linear feature separation, resulting in recall rates below 91.9% on homogeneous datasets like Harvard and RIDER. The random forest model (87.5% [30]), an ensemble learning approach, leverages non-linear decision boundaries to improve robustness against MRI artifacts. Performance also varies across datasets such as BraTS and Figshare, where preprocessing significantly influences results. For instance, K-means clustering detects 94.07% of tumors in the BraTS dataset when region isolation is applied but performs poorly on low-resolution images.

#### 3.1.2. Tumor Segmentation Using Machine Learning

Sharma et al. [32] presented an innovative technique that uses neural networks, OTSU thresholding, and Differential Evolution (DE) to segment brain tumors in MRI data. The steps include image preprocessing, threshold identification using DE, tumor detection using OTSU, and classification of segmented images. On images acquired from 56 patients, this combined technique obtained 94.73% segmentation accuracy. Sharif et al. [33] developed a novel methodology to segment and classify brain tumor inside MRI glioma neuroimages. They improved images with Triangular fuzzy median filtering and segmented the images using unsupervised fuzzy sets. Texture features were the extracted lesion features, where textural features were classified by Extreme Learning Machine and regressional ELM. With the use of BRATS 2012–2015 datasets, 95.7% accuracy was detected using their method.

Zhang et al. [34] suggested a tumor segmentation method using clustering approach that improves stability and reduces noise by utilizing morphological operations and adaptive Wiener filtering. After combining fuzzy C-means and K-means++ based on Gaussian kernels for robust segmentation, they enhanced tumor borders through post-processing, achieving 94.6% sensitivity, 99.41% specificity, and 90.87% recall, on a dataset with 100 images. A three-stage approach for MRI brain tumor segmentation was developed by Atia et al. [35]. To begin with, morphological operations were used for removing the skull to get the brain tissues. Then, the region including lesion was located with the particle swarm optimization (PSO), which was guided by ANOVA. Lastly, K-means clustering classified the pixels in this region as tumor or non-tumor. The method demonstrated a 96% accuracy on KICA and BraTS 2015 datasets, demonstrating its efficacity in diagnosis, detection, and radiotherapy planning for brain cancer. ŞİŞİK et al. [36] presented a methodology using an Extreme Learning Machine (ELM) in conjunction with the fast fuzzy c-means (FRFCM) clustering method to segment brain tumors from MRI. Trained on 3200 MRI images, the model segmented tumors with 98.75% accuracy on TCGA-GBM and REMBRANDT datasets, offering a cost-effective, portable, and highly accurate solution with potential clinical applications in automated tumor detection, treatment planning, and real-time processing. Babu et al. [37] developed a technique that uses clustering (K-means and FCM) and dimensionality reduction (PCA or RPT) to segment tumors from brain MRI images. This method was able to reduce computational complexity and to improve segmentation accuracy, achieving 96.5% accuracy when evaluated on BrainWeb and BRATS 2015 datasets, with potential clinical applications in automated brain tumor segmentation for improved diagnosis, treatment planning, and real-time analysis of brain MRI images. Rajan et al. [38] introduced a method that uses fuzzy c-means (KMFCM), K-means, and an active contour approach for segmentation after preprocessing with noise reduction and picture enhancement. A support vector machine (SVM) was then used to classify the tumor type based on the feature vectors produced by the adaptive gray-level co-occurrence matrix (AGLCM). Alqazzaz et al. [39] developed a segmentation methodology that combinede machine-learned features using SegNet along with the hand-crafted features based on the grey-level co-occurrence matrix (GLCM). By focusing just on the ROI, these techniques may suppress the irrelevant area. They also employed a decision tree classification system to categorize tumor areas into edema, necrosis, and increased tumor. The proposed method produced F-measures of 98%, 75%, and 69% for whole tumor, core, and enhanced tumor segmentation on the BraTS 2017 dataset, respectively. These machine-learning-based approaches for brain tumor segmentation are summarized in Table 3.

Clustering-based methods such as Fuzzy C-Means (FCM) and K-means achieve high accuracy (up to 96% [35]) on structured datasets like BraTS 2015. However, they perform poorly with diffuse tumors, with Dice scores dropping by up to 15% [34], due to their dependence on pixel intensity alone. The combination of support vector machines (SVMs) with morphological operations [39] help address this limitation by incorporating texture features (e.g., Gray-Level Co-occurrence Matrix, GLCM), achieving an F1-score of 98% for whole tumor segmentation. ELM-based models [36] outperform traditional classifiers (achieving an F1-score of 98.75%) by handling high-dimensional feature spaces without overfitting.

#### 3.1.3. Tumor Classification Using Machine Learning

Çınarer et al. [40] proposed a tumor classification methodology using statistical imaging characteristics based on MR image features classified as gliomatosis, multi-focal, multi-centric, and n/a. This study’s primary goal was to assess how well four machine learning algorithms classified these features. The methods under consideration were Linear Discriminant Analysis (LDA), k-nearest neighbors (KNN), random forest (RF), and support vector machines (SVMs). The highest overall accuracy of 90% for tumor classification was obtained using SVMs. A new method for classifying brain tumors based on the k-nearest neighbor (KNN) algorithm was presented by Ramdlon et al. [41], this method might improve the accuracy of diagnosis and treatment planning. This system examines T1 and T2 MRI sections, diagnosing different types of astrocytoma, glioblastoma, and oligodendroglioma on the basis of axial MRI slices. It includes enhancement, binarization, and morphology with watershed to isolate tumor regions. Then, this classification method uses the extracted shape features following segmentation. Their approach showed an accuracy of 89.5%, which can effectively differentiate the types of tumors and support medical decision making in clinical workflows. Marghalani et al. [42] introduced an intelligent classification system for MRI brain images to identify the existence of brain pathologies, including tumors, Alzheimer’s disease (AD), and normal conditions. The algorithm showed good performance in discriminating between MRI images of tumor subjects, Alzheimer’s disease, and healthy individuals with a Bag of Features module. This approach was able to classify tissues with an average accuracy of 97%, indicating it as quite efficient for diagnosing brain pathologies, providing a complementary and faster alternative to manual analysis. This method may provide an effective approach for the clinical classification of brain tumors, leveraging advanced image processing and machine learning techniques to improve diagnostic accuracy and efficiency in medical practice. Rao et al. [43] developed a model to classify tumors using a Normalized Median Filter (NMF), contrast enhancement, segment tumors with binomial thresholding, and extract key features using GLCM and SGLDM. Harris Hawks Optimization (HHO) was used to improve feature selection, while KSVM and SSD classifiers distinguish between benign and malignant tumors, further categorizing malignancies by severity. The model showed accuracies of 99.2%, 99.36%, and 99.15%, when tested on BraTS datasets from 2018 to 2020, respectively. It offered a robust tool for clinicians, enhancing diagnostic accuracy, improving treatment planning, and ultimately contributing to better patient management in oncology. Mandle et al. [44] proposed an automated tumor classification system that includes principal component analysis (PCA) for feature selection, discrete wavelet transform (DWT) for feature extraction, and preprocessing with skull stripping and segmentation using optimal K-means clustering. They classified tumors using a kernel-based support vector machine (K-SVM) to distinguish between benign and malignant instances. The proposed framework showed an accuracy 98.75%, a precision of 95.43%, and a recall of 97.65%, when tested on a 160 MRI images dataset. Kaur et al. [45] suggested a tumor classification methodology based on preprocessing, segmentation, feature selection, and SVM classifier. Preprocessing includes denoising using a median filter variant and alignment of pixel definitions. Segmentation applies high accuracy multi-vector methods, and classification in turn extracts measurable features. They used SVM with RBF and linear kernels, providing an accuracy of 87.5%, outperforming Fine KNN, Boosted Trees, and Bagged Trees, which showed accuracy of 75%, 62.5%, and 75%, respectively. To classify brain tumors from MRI data, Jiang et al. [46] applied five different machine learning models: Stochastic Gradient Descent (SGD), k-nearest neighbors (K-NN), decision tree, support vector machine (SVM), and logistic regression. They employed a dataset comprising 1000 MRI images, encompassing normal, glioma, and meningioma pituitary tumors, and subjected it to preprocessing. This entailed resizing and the application of principal component analysis (PCA) for the purpose of reducing the dimensionality of the data. K-NN achieved the highest accuracy (98.51%), followed by SVM (97.5%), logistic regression (96.13%), SGD (85%), and decision tree (84.19%), highlighting the effectiveness of K-NN and SVM for brain tumor classification. These machine-learning-based approaches for brain tumor classification are summarized in Table 4.

The effectiveness of linear classifiers like support vector machines (SVMs) and Linear Discriminant Analysis (LDA) is largely dependent on the representativeness of extracted features. For instance, SVM achieves 90% accuracy on the well-annotated REMBRANDT dataset [40], but its performance drops to 87.5% on imbalanced datasets, such as those dominated by the meningioma class [46]. In contrast, when combining texture and morphological descriptors, such as Haralick features with the Harris Hawks Optimization (HHO) algorithm, ensemble approaches such as random forest show a greater ability to represent intricate feature interactions, with 99.36% accuracy [43]. Variability between datasets, such as TCIA and BraTS, further highlights the necessity of data augmentation, particularly for kernel-based models; for example, k-nearest neighbors (KNN) shows a performance increase from 89.5% [41] on raw data to 98.51% [46] when trained on augmented samples.

#### 3.1.4. Survival Prediction Using Machine Learning 

Chato et al. [47] presented a survival prediction framework using denoising wavelet transform (DWT) method. They utilized the BraTS dataset to extract histogram features from 163 samples and assess different machine learning models. The highest accuracy was achieved in 10-fold cross-validation using a linear SVM with Daubechies level-4 (db4)-L4 after applying DWT noise reduction and incorporating patient age. Excluding age, a simple decision tree with db2 at levels 1 and 3 (L1, L3) performs best, with db2-L3 achieving 66.7% accuracy in a hold-out validation. This approach underscores DWT’s value in improving prediction through noise reduction. The clinical use of the presented method in predicting overall survival time for brain tumor patients can lead to improved diagnostic accuracy, personalized treatment strategies, and ultimately better patient outcomes. Shboul et al. [48] focused on survival prediction in glioblastoma patients, extracting 1207 texture and other features from MRI scans, evaluating their performance for prognosis, and then incorporating the best features in a random forest regression model. The model was tested on 163 cases from the BraTS17 dataset, and the resulting normalized root mean square error was 30%. In contrast, 10-fold cross-validation yielded an accuracy of 63%. It should be noted that these results pertain to survival outcomes, despite the inherent heterogeneity of the tumor in MRI. In their study, Baid et al. [49] used radiomics features extracted from MRI images of GBM patients to guide personalized treatment planning. They also extended FLAIR and T1ce MRI data by capturing first-order intensity, shape, volume, and texture features, and again, refined the region of interest by using Stationary Wavelet Transform to capture higher-order directional details. Their methodology achieved OS prediction scores of 69.5%, 67.1%, and 55.8%, respectively, for training, validation, and test sets using the BraTS 2018 dataset. Choi et al. [50] investigated whether MRI-based radiomic features can enhance survival prediction accuracy for lower grade gliomas beyond clinical and genetic factors, such as IDH mutational status. From both public and institutional (TCGA/TCIA) datasets, they extract 250 radiomic characteristics from preoperative MRI images of 296 glioma patients. A random survival forest (RSF) model was trained on the institutional dataset and subsequently verified on TCGA/TCIA data. This verification was conducted using the aforementioned characteristics in conjunction with non-imaging variables, namely age, resection extent, WHO grade, and IDH status. Their model identifies 71 significant radiomic features, improving prediction accuracy (iAUC 70.9%) compared to non-imaging data alone (iAUC 62.7%), thus demonstrating that adding radiomic phenotyping to clinical and genomic profiles enhances survival prediction for glioma patients. For glioma patients’ survival prediction, Manjunath et al. [51] analyzed the correlation between clinical data and radiomic features. They employed 3D Slicer for feature extraction and tumor segmentation as well as the assessment of survival prediction algorithms among the selected machine learning techniques. Finally, the KNN model was reported with the highest accuracy (64.4%) using results of clinical and radiomic data from the BraTS 2020 dataset, offering a promising approach for improving OS predictions in tumor patients, leveraging advanced imaging techniques and machine learning to enhance clinical decision making.

Rajput et al. [52] established an end-to-end artificial intelligence model to predict the survival days (SD) of glioblastoma multiforme (GBM) patients. First, they identified tumors in MRI images through segmentation, and then, obtained 1265 characteristics which combine shape-based and location-based and radiomics features and patient metadata. They employed recursive feature elimination alongside permutation importance and correlation analysis for feature selection process which yielded 29 main features. These include age, key tumor locations, and radiomics characteristics. The model achieves better interpretability because post-hoc analysis identifies patterns which match clinical knowledge such as survival risks for patients above 50 with central brain tumors. The proposed model achieved an SD accuracy rate of 53.8% for training and 55.2% for validation, showing a 33% improvement over existing BraTS-2020 benchmarks and highlighting its potential for clinically relevant survival prediction. These machine-learning-based approaches for brain tumor survival prediction are summarized in Table 5.

The current application of traditional parametric models for survival prediction exhibits low efficiency, reaching 55% C-index [49] due to their inability to handle the complex correlations among radiomic features. The regularized prediction model Lasso-Cox regression reaches a 66.7% C-index [47] due to its effectiveness in feature selection. Simultaneous use of random forest models with clinical variables showed an integrated AUC (iAUC) of 70.9% ([50]). However, these models often remain limited by small sample sizes (e.g., *n* = 296). More recent ensemble approaches have shown promise, with models achieving 74% accuracy on the BraTS 2020 dataset [52] by integrating diverse indicators, including tumor location and shape descriptors.

### 3.2. Deep Learning Techniques

The application of deep learning methods in MRI-based brain analysis has become one of the advanced non-invasive technologies reshaping various tasks, including detection, characterization of abnormal brain structures, classification, and prediction of survival. In contrast to the previously suggested approaches, deep learning models may be trained to automatically and without supervision learn complex and hierarchical features straight from MRI data, requiring little feature extraction or detection. This capability is especially useful since brain imaging is very variable and intricate as small patterns in the structural formation are often essential in making prognosis and diagnosis. The findings demonstrate that by collecting both high-level representations and low-level aspects of MRI data, deep learning holds significant potential for improving the accuracy and reliability of brain analysis processes.

#### 3.2.1. Tumor Detection Using Deep Learning 

A robust methodology for the identification of brain cancers in MRI images was presented by Musallam et al. [53], comprising a distinctive deep convolutional neural network (DCNN) configuration and a three-stage preprocessing methodology. The suggested DCNN, which uses batch normalization, a lightweight design with few layers, and larger 7 × 7 convolutional filters, classifies MRI images as glioma, meningioma, pituitary, or normal. This methodology showed a detection accuracy of 98.22% on a 3394 MRI dataset, improving brain tumor detection accuracy and efficiency and supported clinicians in making informed decisions, ultimately enhancing patient outcomes. Md Ishtyaq et al. [54] suggested using MRI data to effectively identify brain cancers using a convolutional neural network (CNN) model. When compared to other models, such as Inception V3, ResNet-50, and VGG-16, the suggested CNN model performed better. After training on a dataset of 3264 preprocessed and improved MR images, it obtained an accuracy of 93.3%, an AUC of 98.43%, a recall score of 91.19%, and a loss score of 25%. In order to identify and classify brain tumors based on MRI, Nayak et al. [55] created a convolutional neural network called Dense EfficientNet that uses min-max normalization and data augmentation. Using this technique, 3260 T1-weighted contrast-enhanced brain MRIs that were divided into pituitary, glioma, meningioma, and no tumor classes have been distinguished. This model, a variant of EfficientNet with additional dense and drop-out layers, was found to perform exceptionally well, achieving an accuracy of 99.97%, and 98.78%, respectively, for training and test, with an F1-score of 98% during testing. It provides notable clinical advantages, such as improved detection accuracy, automation in tumor identification, assistance in clinical decision making, and early tumor detection. Obeidavi et al. [56] presented an automated brain tumor detection system based on a residual convolutional neural network architecture. Their method uses a deep network with 11 long skip-connections to handle challenges such as disappearing gradients and learn features at many scales. The model achieved an accuracy of 94.43%, a mean IoU of 54.21%, and a weighted IoU of 93.64% on the BraTS 2015 dataset. A convolutional neural network (CNN)-based model for multi-class brain tumor detection was developed by Mahjoubi et al. [57], using ReLU and SoftMax activations, this model was made up of five convolutional layers with filters ranging from 32 to 512, five max-pooling layers, a flattening layer, and dense layers with 128 and 4 units, respectively. With an accuracy of 95.44%, a recall score of 95%, and an F1-score of 95.36% on a combined dataset from Figshare, SARTAJ, and Br35H, the model shows its efficacy in accurately recognizing and categorizing brain cancers for early diagnosis and treatment. Hashan et al. [58] suggested a convolutional neural network (CNN) approach that is optimal for brain MRI tumor identification, where the Adam algorithm was then used to optimization. Tested on the 400-image dataset, this model showed an accuracy of 90% and a F-score of 89%, which was better than the previous methodologies like SVM and MFDFA + random forest with accuracies of 81.47% and 86.7%, respectively. Bhanothu et al. [59] suggested a Faster R-CNN approach with a VGG-16 network to tumor detection and segmentation in MRI images with glioma, meningioma, and pituitary tumors. Their method only uses bounding boxes to detect and categorize tumors, achieving accuracies of 75.18%, 89.45%, and 68.18% respectively, for gliomas, meningiomas, and pituitary tumor, and an overall mean average precision of 77.60% on a dataset with 233 patients.

A new, lightweight architecture of the U-Net model, named LeU-Net, was presented by Rai et al. [14]; this model was designed for fast and accurate tumor detection in brain MR images. LeU-Net was derived from the Le-Net and U-net models, but restructured to have less of a structure. It was proposed for faster processing time and a higher accuracy rate on small sets of data. The model was tested on 253 MRI images, containing both cropped and uncropped images, reaching 98% on the cropped images and 94% on the uncropped images. Mohan et al. [60] proposed a technique that uses the U-Net CNN model to detect and categorize brain cancers. Their strategy incorporated the functional capability of MRI imaging to acknowledge the tumor regions and employed it to detect the early stages to eliminate severe progress. The proposed U-Net CNN model was very effective, with 98.67% accuracy, 96.72% sensitivity, and 94.86% specificity scores on a 3264 MRI images dataset. This model can be used clinically for early brain tumor detection, accurate diagnosis, and treatment planning.

Aamir et al. [61] suggested an automated method for MRI image-based brain tumor identification. Their methodology starts with processing brain MRI scans to improve visual quality. Then they used two distinct pretrained deep learning models to extract useful information from the image. A hybrid feature vector was created by combining the acquired feature vectors using the partial last squares (PLS) method. The top tumor sites were then determined using agglomerative clustering. Using a dataset of 233 patients’ MRI pictures, the suggested model achieved a 98.95% accuracy rate. Almadhoun et al. [62] applied deep learning algorithms for tumors detection and classification in brain MRI scans as primary or metastatic. Numerous deep learning architectures were evaluated, such as InceptionV3, VGG16, ResNet50, MobileNet, and their model. The efficacity of these methods for automated brain tumor detection was demonstrated by the models’ F1- score accuracies of 98.28% (custom model), 99.86% (VGG16), 98.14% (ResNet50), 88.98% (MobileNet), and 99.88% (InceptionV3) on 10,000 brain MRI images dataset. Abdusalomov et al. [63] developed a refined YOLOv7 model to accurately detect brain tumors in MRI scans. They enhanced feature extraction with a convolutional block attention module (CBAM), detected small tumors using a spatial pyramid pooling fast+ (SPPF+) layer, and fused multi-scale feature using a bi-directional feature pyramid network (BiFPN). The authors demonstrated that refined YOLOv7 achieves a high accuracy of 99.5% on a dataset with 10,288 images. Taher et al. [64] presented BRAIN-TUMOR-net, a custom convolutional neural network model based on transfer learning for brain tumor detection and classification. A comparison of the proposed model with other pretrained deep learning models, such as ResNet50, InceptionResNetv2, and Inceptionv3, reveals that the former exhibits the highest level of accuracy, with values of 100%, 97%, and 84.78% on three MRI datasets, respectively. To identify brain cancers in MRI images, Khaliki et al. [65] used a custom multi-layer CNN in addition to a few CNN-based transfer learning models, such as InceptionV3, VGG16, VGG19, and EfficientNetB4. The VGG16 transfer learning model surpassed the custom multi-layer CNN, which had 91.3% accuracy on a dataset of 2870 MRI images, with 98% accuracy, 97% F1-score, 99% AUC, 98% recall, and 98% precision.

Krishnan et al. [66] developed a novel Rotation Invariant Vision Transformer (RViT) architecture for tumors detection from brain MRI images. Generating rotation-invariant patch embedding involves turning the input MRI pictures and adding special rotation embeddings. The RViT architecture consists of splitting the image into patches, applying rotation and positional embeddings, and using a transformer encoder to process and predict tumors with a classification head. It achieved results of 100% sensitivity, 97.5% specificity, 98.4% F1-score, 97.2% Matthew’s Correlation Coefficient (MCC), and 98.6% total accuracy on 7023 brain MRIs dataset from Figshare, SARTAJ, and Br35H datasets. Clinically, the RViT model can enhance healthcare professionals’ ability to diagnose and treat brain tumors, improving patient care and outcomes. Asiri et al. [67] developed FT-ViT, a fine-tuned vision transformer, to efficiently and precisely identify brain cancers in MRI images. The FT-ViT model divided MRI images and applied self-attention mechanisms to identify healthy and malignant regions. The model was fine-tuned on the CE-MRI dataset after being trained achieving an accuracy of 98.13%. Poornam et al. [68] presented a brain tumor detection model developed called VITALT, which integrates a Vision Transformer (ViT), Linear Transformation Module (LTM), Split Bidirectional Feature Pyramid Network (S-BiFPN), and soft quantization all taken to intensify the MRI image feature maps. Tested on four different datasets, VITALT obtained good classification rates of 99.08%, 98.97, 98.82%, and 99.15%. It represents a promising advancement in medical imaging, with the potential to improve the accuracy and efficiency of brain tumor detection in clinical practice. These deep-learning-based approaches for brain tumor detection are summarized in Table 6.

Transformer-based models, such as RViT, which use complete attention processes, perform substantially better in these settings, with accuracy reaching up to 98.6% [66]. Meanwhile, U-Net architectures continue to excel in segmentation tasks by using skip connections to maintain spatial information, achieving 98.67% accuracy, which is far higher than that of object identification models such as Faster R-CNN, which only achieve 77.6% accuracy [59]. On the other hand, InceptionV3 exhibits a sharp 20% decline in performance when applied to external datasets, highlighting the need for training models with contrast-specific and domain-adaptive data.

#### 3.2.2. Tumor Segmentation Using Deep Learning 

Ngo et al. [69] developed a segmentation method that enhances accuracy for brain tumors of all sizes, especially small ones, by combining dilated convolution for multi-scale feature extraction with multi-task learning. This lightweight approach uses an auxiliary feature reconstruction task to retain small tumor features and improves segmentation without relying on complex, multi-resolution networks. Using the BraTS 2018, this technique obtained a global accuracy of 85.87%. Ranjbarzadeh et al. [70] designed a tumor segmentation technique utilizing a cascade convolutional neural network (C-CNN) and distance-wise attention (DWA). The method of C-CNN extracts the local and global features, while DWA boosts the segmentation by focusing in particular on a tumor central area. With just little differences in ranking top performers, the model achieved Dice scores of up to 92.03% for complete tumors on the BRATS 2018 dataset and between around 91% and 97% over enhancing/wall-core overlap classes/active tumor cores via all competitors. For brain tumor segmentation, Fidon et al. [71] used an optimized 3D U-Net-based architecture that included a population-wise generalized loss function, optimizer, and per-sample loss function sampling. Multi-class segmentation of brain tumors requires a loss function that can utilize the context provided by region hierarchies to distinguish tumor sub-regions better than generalized Dice. The model achieved a mean Dice score of 88.9%, 84.1%, and 81.4%, respectively, for whole tumor, tumor core, and enhancing categories on the BraTS 2020 Challenge. Ottom et al. [72] created the innovative Znet deep learning framework for tumor segmentation from 2D brain MRIs. The Znet neural network implemented adversarial networks, as well as the structure of U-Net, including an encoder to downsample the image and a decoder to upsample, which contained 5 blocks in each of them to provide skip connections that helped the data retain spatial information. The final layer of blocks defines the segmentation mask. Trained on the TCGA-LGG dataset, the model achieved 96% mean Dice similarity and 92% Dice similarity, with 99.6% pixel accuracy and 81% F1 score correlation. The Znet model represents a promising development in AI for medical imaging that might greatly increase the precision and effectiveness of diagnosing and treating brain tumors. Rajendran et al. [73] proposed a unique automated deep learning architecture-based technique for segmenting brain tumors in MRI data. They used a three-dimensional convolutional neural network (CNN) and a three-dimensional U-Net model to build their network. This method showed excellent performance for the entire tumor (accuracy 99.40%, precision 98.46%, F1-score 90.27%) and high accuracy in the enhanced part of the tumor (accuracy 100%, precision 95%, sensitivity 93%) on the Figshare MRI dataset, demonstrating that the use of CNNs significantly improved the accuracy of brain tumor segmentation and highlighting their effectiveness in biomedical image segmentation tasks. In order to improve diagnosis time and make diagnostic output more relevant, Mostafa et al. [74] designed a method for segmenting brain tumors using an end-to-end deep learning technique. They achieved this by employing 3D U-Net base convolutional neural networks (CNNs). Using the BraTS dataset, the model achieved 98% accuracy, demonstrating its effectiveness in tumor segmentation and identification in medical imaging and providing a robust foundation for future advancements in the field. A convolutional neural network (CNN) algorithm was created by Vavekanand et al. [75] to segment brain tumors in MRI data. The CNN model included a Sigmoid activation function to predict the presence of tumors, convolutional layers for feature extraction, max pooling for dimension reduction, dropout layers to minimize overfitting, and dense layers for data classification. On a dataset of 1000 scans, it achieved accuracies of 91% and 89% for validation and testing, respectively. Liao et al. [76] presented a tumor segmentation method using a modified Swin-UNet, named Swin-UNet-EPA. This model uses the Swin Transformer as an encoder for feature extraction at various spatial resolutions and U-Net architecture to incorporate these features with high-resolution maps, which ensures detailed segmentation. Highlighting this work, they introduce the EPA (Efficient Paired Attention) block, which fuses both spatial and channel attention mechanisms with a parameter-sharing design to economize overall computational cost but also balanced allocation of attention weights. The suggested model obtained 91.5% Dice coefficient and 84% IoU on the BraTS 2021 dataset. To accurately segment brain tumors from MRI, Cirillo et al. [77] developed a 3D volume-to-volume Generative Adversarial Network (GAN) named Vox2Vox. The proposed Vox2Vox processes multi-channel 3D MRI inputs to segment tumor regions, including the whole tumor, core, and enhancing tumor, achieving mean Dice scores of 87.20%, 81.14%, and 78.67%, respectively, on the BraTS 2020 challenge dataset. After ensembling 10 Vox2Vox models with 10-fold cross-validation, it reached Hausdorff distance 95th percentiles of 6.44 mm, 24.36 mm, and 18.95 mm for these tumor regions on the BraTS testing set. These deep-learning-based approaches for brain tumor segmentation are summarized in Table 7.

Three-dimensional U-Net models have become a go-to solution for brain tumor segmentation in the BraTS dataset, largely because they excel at capturing the spatial relationships within 3D MRI scans, achieving strong performance (Dice score: 88.9% [70]). However, their performance can suffer when the data shifts—for example, from MRI to CT scans—often leading to over-segmentation. To address this limitation, a newer approaches have been emerged, including adaptive loss functions, such as the Wasserstein Dice loss [71], which help models to focus more precisely on challenging regions; mechanisms such as those in Swin-UNet-EPA [76], which improve sensitivity to ambiguous tumor boundaries; and Generative Adversarial Networks (GANs), such as Vox2Vox [77], which provide highly realistic segmentations (Dice score: 87.2%), but at the expense of time-consuming and resource-intensive training.

#### 3.2.3. Tumor Classification Using Deep Learning 

Deepak et al. [78] developed a deep-transfer-learning-based brain tumor classification method for the generation of classifier models, which provided significant findings. They started with a pretrained GoogLeNet model on ImageNet, and finetuned its final layers for a 3-class brain tumor classification task based on the Figshare dataset. In order to perform classification using techniques like the k-nearest neighbor (KNN) algorithm, the support vector machine (SVM), or the SoftMax layer within CNNs, the system must learn the deep convolutional neural network (CNN) features extracted from the background of brain MRI images. With an average classification accuracy of 98% during a five-fold cross-validation on a patient-level, the results showed that the suggested strategy performed better than current state-of-the-art techniques. Kumar et al. [79] presented a comprehensive methodology for diagnosing and classifying brain tumors based on MRI imaging, utilizing publicly available datasets. The CNN model, which incorporates the transfer learning concept and pretrained models such as ResNet-50, VGG-16, and U-Net, has demonstrated promising results following fine-tuning for superior image segmentation. The model showed a precision of 93.56% and recall of 92.19% for benign tumors and a precision of 95.45% and recall of 92.45% for malignant tumors, with F1-scores of 92.33% and 93.92% for benign and malignant tumors, respectively, on a dataset of 1572 T1w MRI images. Using five state-of-the-art architectures (InceptionResNetV2, DenseNet121, ResNet152V2, Xception, and DenseNet201), each with a deep dense block and SoftMax output layer, Asif et al. [80] developed a deep-learning-based method for brain tumor classification in MRI, primarily using transfer learning techniques. After fine-tuning on the Figshare dataset, they were able to achieve accuracies of 99.67% for three-class and 95.87% for four-class classifications using Xception. The suggested model provides radiologists with an automated medical diagnostic system that facilitates fast and precise clinical decision making. Haq et al. [81] developed an extensive technique based mainly on convolutional neural networks (CNNs). They proposed two convolutional neural network (CNN) architectures. On the BraTS2018 dataset, the first one classified glioma, meningiomas, and pituitary tumors with a Dice Similarity Coefficient (DSC) of 95.8% and an accuracy of 97.3%, while the second one obtained a DSC value of 94.3% and an accuracy of 96.5%. A novel CNN technique to distinguish between glioma, pituitary, and meningioma tumors in brain MR images was introduced by Rasheed et al. [82]; it was composed of several layers, each of which combines convolutional, batch normalization, dropout, max pooling, global average, and dense layers with L1/L2 regularization. For classification, they used SoftMax activation, and it was trained with the Adam optimizer and ReduceLROnPlateau callbacks. This method demonstrated a 98.04% classification accuracy on a dataset of 3064 MRIs. A 25-layer convolutional neural network (CNN) model was developed by Kumar et al. [83] with the objective of enhancing the classification of brain tumors. Multiple convolution layers were applied to extract abstract hierarchical features in their CNN architecture. The model demonstrated an accuracy of 86.23% when utilizing the Adam optimizer and 81.6% when employing the Sadam optimizer on a dataset comprising 3064 T1-weighted MRI images. Joshi et al. [84] introduced a new deep learning approach for the precise segmentation and classification of brain tumors. The developed model got classification accuracies of 97. 8% and 96.9% using the ResNet-50 and ResNet-101 structures, respectively, on the BRATS 2020 dataset. To improve the model’s capacity for generalization, they used transfer learning approaches to improve the feature extraction and augmentation process, such as flipping and rotation. Then, to achieve precise tumor delineation, they used deconvolutional layers and skip connections in the U-Net architecture for tumor segmentation. With a high Dice Similarity Coefficient (DSC) of 98.5%, the suggested model performs better than earlier models, showing substantial gains in segmentation and classification accuracy while cutting down on computation time.

To enhance tumor classification from brain MRI images, Haque et al. [85] created the NeuroNet19 deep learning model, which is based on the VGG19 model. The proposed model includes an inverted pyramid pooling module (iPPM) to improve features extraction and capture multi-scale features. NeuroNet19 achieved an accuracy of 99.3% on 7023 MRI images dataset. This model can support radiologists and oncologists by enabling early detection and accurate classification of various brain tumors, improving diagnostic efficiency and patient outcomes. Mohanty et al. [86] presented a unique deep learning technique based on a convolutional neural network (CNN) and a soft attention mechanism for improving tumor classification from brain MRI data. They designed a novel CNN with four layers where features were collected from all the layers to form a feature vector. This was followed by the soft attention mechanism which concentrated on the most relevant features, enhancing decision making. This model showed a strong performance in identifying tumor types by its ability to classify tumors into pituitary, meningioma, and glioma tumors, as it achieved high precision (95.57%, 94.61%, 95.16%), recall (93.64%, 96.88%, 94.65%), and F1-scores (94.45%, 95.98%, 95.00%) for each type, respectively, and a specificity of 87.41%, on the Figshare dataset. Jain et al. [87] created an ensemble deep-learning method (EDL-BTC) for the early diagnosis of brain tumors using the BRATS dataset using transfer learning. They used ResNet50, InceptionV3, and MobileNetV2 to extract features from tumor pictures. A ReLU layer and Dense Layer were applied as classifiers, with accuracy validated through 5-, 10-, and 20-fold cross-validation. The model achieved accuracies of 98.3%, 98.6%, and 98.6% across the three cross-validation settings, outperforming advanced pretrained models. The EDL-BTC can significantly enhance clinical workflows by assisting radiologists and oncologists in the early detection and accurate classification of brain tumors, ultimately improving patient outcomes.

Saboor et al. [88] proposed the Attention-Gated Recurrent Unit (A-GRU) approach for brain tumor classification, which captures both spatial information within MRI images and the temporal evolution of tumor characteristics. The integration of GRU sequential modeling features with attention mechanisms allows the model to display which features are most important to its predictive process. Using the BTD dataset, the A-GRU model demonstrated a better performance than traditional CNNs and recurrent networks by achieving a 99.32% classification accuracy during benchmarking. This high performance demonstrates the model’s effectiveness for accurate and interpretable brain tumor diagnosis in E-healthcare applications. Özkaraca et al. [89] developed their own CNN-based model for brain tumor classification since they found limitations in current image processing approaches. The researchers tested a simple CNN architecture initially and found performance issues due to its shallow depth. Then, they applied VGG16 and DenseNet with transfer learning, yet discovered that their success in different domains failed to enhance performance in medical image analysis. Consequently, they built a deep CNN model which packed densely arranged layers before training it directly from scratch using a dataset of 7000 MRI images split into Glioma, Meningioma, Pituitary, and No Tumor classes. The model was trained on 80% of the data and tested on 20%, achieving an accuracy between 94% and 97%. Although the model demonstrated strong classification performance, its main limitation was the long processing time due to the dense architecture. These deep-learning-based approaches for brain tumor classification are summarized in Table 8.

The use of deep transfer learning models like ResNet-50 demonstrates strong potential in brain tumor classification tasks, achieving up to 97.8% accuracy [84], and generally outperforming custom-built architectures such as ConvNet (86.23% [83]). The rarity of gliomatosis tumors poses a challenge to these models, due to limited representative samples. Attention-based models, such as NeuroNet19 [83], help overcome this challenge by directing focus toward critical regions like necrosis and edema, achieving even higher accuracy (99.3%). The importance of image resolution is demonstrated by the differences in performance between datasets: lightweight CNNs are adequate for standardized datasets, whereas deeper architectures (e.g., DenseNet201 [80]) perform well on high-resolution MRIs.

#### 3.2.4. Survival Prediction Using Deep Learning

Ben Ahmed et al. [90] created an automated technique for predicting survival time in patients with glioblastoma by using an ensemble of three-dimensional deep convolutional neural networks (CNNs) to MRI data. By using ensemble snapshots of the CNN models, their method addresses the deficiency of tagged medical images and improves the prediction of tumor location using multi-sequence MRI images (FLAIR, T1CE, and T2). This kind of classification divides glioblastoma patients depending on their survival span as a short span and a long span group. On the original BraTS dataset containing 163 training and 46 test samples, the accuracy of their model on unseen test data was 74%. The proposed model has the potential to significantly impact clinical decision making for high-grade glioma patients by providing accurate survival predictions, facilitating personalized treatment approaches, and addressing data limitations in medical imaging. Using MRI scans and clinical data, Jalalifar et al. [91] created a new DL model to forecast both local control and local failure in patients receiving stereotactic radiation treatment for brain metastases. Their architecture focuses on a base InceptionResNetV2 network to extract features from individual MRI slices and then fused by either recurrent or transformer networks, accounting for the spatial relationships between slices. A prediction difference analysis visualization highlights key MRI regions contributing to predictions. When integrating MRI features, clinical factors, and inter-slice dependencies (using an LSTM network), the model, which was tested on 25 test patients (40 lesions) and trained on data from 99 patients (116 lesions), obtained an AUC of 86%. This result demonstrates that the model outperforms those based on clinical variables alone. To create a synthetic post-contrast T1-weighted MRI from precontrast MR sequences, Preetha et al. [92] trained deep convolutional neural networks (dCNNs) based on 500 paired images of glioblastoma patients across multiple institutions and validated it 81.8% with real post-contrast images, C-index values were similar for predictions made using the synthetic (66.7%) and true post-contrast sequences (67.3%), as were median times to progression, suggesting that synthetic MRIs can successfully be used in glioblastoma prognosis. A deep learning encoder–decoder ConvNet model was created by Banerjee et al. [93] for tumor segmentation and OS prediction in glioma patients. Tumors were segmented in three planes (coronal, sagittal, and axial) and fused into a three-dimensional volume. Spatial-pooling, unpooling, and shortcut connections were used to improve detail recovery and boundary preservation, achieving an OS prediction accuracy of 54% on the BraTS 2018 dataset. The proposed model enhances the efficiency and accuracy of brain tumor assessment and management, ultimately contributing to improved patient outcomes. Islam et al. [94] created a three-dimensional U-Net based on attention-CNN for the purpose of segmenting and predicting the survival of brain tumors in MRIs. For survival prediction, they combined radiomic features (e.g., tumor shape, location) and clinical data (e.g., age) to estimate overall survival (OS). Key features like necrosis region shape and location were essential for accurate predictions. The model achieved OS prediction accuracies of 48.3% and 38.3%, respectively, on the validation set and the BraTS 2019 dataset. These deep-learning-based approaches for brain tumor survival prediction are summarized in Table 9.

Three-dimensional convolutional neural networks (CNNs), such as 3D U-Net [94], have demonstrated a stronger ability to capture the spatial complexity and heterogeneity of brain tumors compared to 2D models, achieving 48.3% accuracy in survival prediction tasks. However, this gain in spatial modeling often comes at the expense of interpretability, making clinical adoption more difficult. Integrating radiomic features into deep learning frameworks offers a promising path toward improving prognostic models, such as the deep CNN (dCNN) presented by [92], which obtained a concordance index (C-index) of 66.7%. However, these approaches are heavily dependent on accurate tumor segmentation, which can be challenging in clinical settings. Short-term survival prediction remains particularly difficult, with some models reporting only 38.3% accuracy [94], highlighting the aggressive and unpredictable nature of high-grade gliomas and emphasizing the urgent need for longitudinal and multi-modal data to improve predictive performance.

### 3.3. Hybrid Techniques

A potential method for addressing the challenges of MRI brain tumor analysis (detection, segmentation, classification, and patient survival) is the combination of machine learning and deep learning. Hybrid techniques provide an intermediate approach to combine the feature extraction ability of machine learning algorithms and the hierarchical feature learning of deep learning models. These methods are effective in combining domain knowledge and adaptive learning in order to obtain significantly better understanding of the features in the MRI of the brain. It offered nuanced analysis of both global structures and localized abnormalities, demonstrating significant potential in bridging gaps and achieving superior performance in various brain analysis tasks.

#### 3.3.1. Tumor Detection Using Hybrid Techniques

A hybrid convolutional neural network (CNN) was suggested by Sajid et al. [95] to detect brain tumors in magnetic resonance (MR) pictures. The efficacy of sophisticated regularization strategies including dropout and a unique two-phase training methodology was investigated and validated. To improve the model’s performance, they also incorporated two-path and three-path networks into their suggested hybrid model. On the BraTS 2013 dataset, the proposed model obtained a Dice score of 86%, a sensitivity of 86%, and a specificity of 91%. A training dataset of 3264 brain MR images was used by Saeedi et al. [96], who designed a system to identify tumors. They used a 2D CNN and a convolutional auto-encoder network, respectively, and achieved training accuracies of 96.47% and 95.63%. The 2D CNN, including four pooling and eight convolutional layers, was optimal, and it gave an average recall of 95%; moreover, the ROC curve almost reached 1. The KNN accuracy was arbitrarily determined to be at 86% as the highest accuracy compared to the tested machine learning methods. Last of all, they concluded that the 2D CNN is suitable for clinical use because the high accuracy and the low complexity. Anantharajan et al. [97] suggested a novel method for detecting brain tumors in MRI images that is based on deep learning and machine learning. The MRI images were initially resized to a uniform size and underwent preprocessing, which involved the utilization of a medium filter and an adaptive contrast enhancement algorithm (ACEA). The preprocessed pictures were then divided using segmentation based on fuzzy C-means (FCM). The grey-level co-occurrence matrix (GLCM) method was used to extract features such as energy, mean, contrast, and entropy. The abnormal tissue was then categorized using the suggested Ensemble Deep Neural support vector machine (EDN-SVM) classifier. The model’s accuracy, sensitivity, and specificity scores for distinguishing between abnormal and normal tissue in MRI brain scans were 97.93%, 92%, and 98%, respectively, using a dataset of 255 T1-mode MRI images. Abdusalomov et al. [98] proposed a hybrid tumor segmentation method, combining the YOLOv5 object detector with non-local neural networks (NLNNs). A substantial corpus of MRI data pertaining to the human brain was procured from a multitude of sources, and the YOLOv5 algorithm was then refined to enhance its capacity for tumor detection. To enhance detection performance, they added new modules including K-means+, Spatial Pyramiding Pooling Fast+ (SPPF+), and Bi-directional Feature Pyramid Net (Bi-FPN). The YOLOv5 and NLNNs together obtained 86% and 83% recall rates, respectively. Jahangir et al. [99] created a system for MRI scan brain tumor detection that combines vision transformer (ViT) models with convolutional neural network (CNN) based transfer learning classifiers. They created six CNN-based classifiers (EfficientNetB0, DenseNet201, ResNet152V2, InceptionV3, Xception, and MobileNet) in addition to four ViT models (ViT-L/32, ViT-B/32, ViT-L/16, and ViT-B/16). Using an MRI dataset of 7023 images, ViT models achieved better performance on training data (100% precision, 100% recall, 100% F1-score) while CNN-based models achieved better performance on test data (98.233% precision, 98.271% recall, 98.234% F1-score), indicating the efficacy of their methodology. In order to identify brain malignancies in MRI images, Khairandish et al. [100] proposed a hybrid deep learning method that combines an SVM classifier with Faster R-CNN. This model’s goal is to locate tumor areas and classify them as either benign or malignant. The goal of this is to enhance conventional machine learning techniques that depend on manually created features and professional analysis. The proposed model, implemented via OpenCV, demonstrated a 98.81% accuracy rate on the BraTS 2015 dataset. It has the potential to significantly impact clinical practices in neuro-oncology by improving the accuracy and efficiency of brain tumor detection and classification.

Sahoo et al. [101] introduced a new detection model that fuses generative adversarial networks (GANs) together with modulated convolutional neural networks (CNNs) to boost early brain tumor diagnosis accuracy. A hybrid GAN ensemble performed preprocessing on brain tumor MRI images to increase data diversity and robustness. Then, a customized CNN model was used to classify the augmented images. A soft voting mechanism was used to generate final predictions, selecting outputs from the GAN that achieves the highest performance metrics. Using a dataset of 3064 CE-MR images and among the evaluated GAN variants, the Progressive Growing GAN (PGGAN) delivered the best results, achieving an accuracy of 98.85%, along with high precision (98.45%), recall (97.2%), F1-score (98.11%), and a low latency of 3.4 s. This hybrid approach demonstrated strong potential for real-time, accurate brain tumor detection, offering a reliable aid for clinicians in early-stage diagnosis. Mathivanan et al. [102] employed deep transfer learning techniques to enhance brain tumor detection accuracy using MRI images. They evaluated four pretrained architectures—ResNet152, VGG19, DenseNet169, and MobileNetv3—by fine-tuning them on a labeled brain tumor dataset of 7023 MRI images, comprising four classes: pituitary, normal, meningioma, and glioma. Then, image enhancement methods were used to address data imbalance issues and improve model performance. The models were trained and validated using five-fold cross-validation, ensuring robustness. Among the architectures tested, MobileNetv3 achieved the highest accuracy of 99.75%, highlighting the effectiveness of transfer learning in medical image classification tasks. These hybrid techniques for brain tumor detection are summarized in Table 10.

CNN-SVM hybrid models efficiently merge local pattern detection capabilities of deep architectures like Faster R-CNN with the outlier robustness of support vector machines (SVMs), achieving an accuracy of 98.81%. Using the BraTS 2015, this combination better performance than models that are purely deep learning based, especially in the presence of noisy data. Furthermore, GAN-augmented frameworks such as the Progressive Growing GAN (PGGAN) [101], which attained an accuracy of 98.85%, enhance model generalization by generating diverse synthetic data. However, these models may accidentally produce artifacts in non-pathological (healthy) areas, which might impact downstream clinical interpretation.

#### 3.3.2. Tumor Segmentation Using Hybrid Techniques 

Thillaikkarasi et al. [103] suggested combining a kernel-based convolutional neural network with a multi-class support vector machine (M-SVM) to automatically segment malignancies in brain MR images. The technique starts with preprocessing, which includes applying Contrast Limited Adaptive Histogram Equalization (CLAHE) and Laplacian of Gaussian (LoG) filtering on MRI images to improve picture quality and create a more uniform intensity distribution. Subsequently, key features related to the shape, position, and surface of the tumor are extracted. Then, they used M-SVM for feature classification and kernel-based CNN for tumor segmentation. This approach achieved an accuracy of 84% on a dataset of 40 MRI images. Daimary et al. [104] suggested architectures that include SegNet, U-net, and ResNet18 architectures in a hybrid format. The U-SegNet and Seg-UNet models integrate SegNet’s efficiency with U-Net’s skip connections, differing in depth, while Res-SegNet combines SegNet with ResNet18 using element-wise addition for skip connections. On the BraTS dataset, these models’ relative accuracies for SegNet3, SegNet5, U-Net, U-SegNet, Res-SegNet, and Seg-UNet were 97.62%, 98.19%, 98.08%, 98.24%, 98.85%, and 99.11%, respectively, suggesting that hybrid models performed better than standalone models.

Five topologies from the “U-Net (modified)” were proposed by Munir et al. [105] to segment tumors from brain MRIs. On the BraTS-2019, the proposed method achieved Dice Coefficients of 72.3%, 79.8%, 83.13%, 82.01%, and 87.75%; sensitivities of 69.7%, 93.61%, 72.67%, 73.26%, and 90.26%; and specificities of 87.2%, 94.29%, 99.92%, 99.8%, and 99.42%, respectively, for Baseline U-net, Recurrent-inception U-net (MI-Unet), Depth-wise Separable MI-Unet, Hybrid Model, and Depth-wise separable Hybrid model.

Balamurugan et al. [106] presented an enhanced LuNet classifier for tumor classification as either glioma or meningioma, as well as a novel approach to segment and classify brain tumors utilizing the hybrid deep convolutional neural network (DCNN). Their approach used VGG16 to perform the feature extraction step then extracting 13 categorical features and classifying it with the extended LuNet algorithm. Using 253 MR images as a dataset, the method achieved an accuracy of 99.7%, demonstrating superior performance to that of conventional algorithms, including random forest, ResNet-50, SVM, decision tree, GoogLeNet, and AlexNet. To categorize and segment brain tumors caused by glioblastoma, Sahli et al. [107] created a hybrid model dubbed ResNet-SVM. ResNet was used to address gradient diffusion problems and extract deep feature efficiently, and it enables quicker training and increases the model’s accuracy. Features were then grouped into tumor types using SVM to improve classification accuracy of necrosis, edema, and enhancement regions. The method was trained and validated with MRI data consisting of 260 training cases and 112 validation cases, it yielded scores of 89.36%, 92.52%, and 90.12% respectively, for accuracy, specificity, and precision. Ramamoorthy et al. [108] suggested a new hybrid method named TransAttU-Net for tumor segmentation from brain MRIs, which utilizes the transformer-based attention mechanism with a U-net model. TransAttU-Net segmented tumor regions using multi-scale skip connections and multi-level directed attention. Evaluated on BraTS 2019 and 2020 datasets, this method produced an average Dice Similarity Coefficient of 98.87% along with 98.26% specificity, 98.85% recall, and 98.47% accuracy; this technique could be used to improve the accuracy and efficiency of medical image segmentation in real clinical scenarios. To accurately separate gliomas in MRI images, Shedbalkar et al. [109] suggested a hybrid method that integrates transformer-based modules with the UNet architecture. They used the encoder–decoder architecture of UNet to exploit spatial hierarchies and incorporated the Swin Transformer module to capture the global context. The hybrid attention mechanism calculates attention at local and global levels, leading to a better feature selection for the particular task. On the BraTS datasets (2015, 2017, 2019, 2020, and 2021), this suggested model reached Dice scores of 94%, 92.1%, 83%, and 94%, respectively.

Almufareh et al. [110] showed the promise of deep learning methods for brain tumor segmentation in MRI using a novel approach based on the YOLO framework. Since the complexity of the presented process requires standardizing the methodology, a deep learning method was implemented. Two YOLO architecture types, YOLOv5 and YOLOv7, were used to achieve precision scores of 93.6% and 93.5% and recall scores of 90.6% and 90.3%, respectively, using the Figshare dataset. Datta et al. [111] suggested a normalization preprocessing combined pixel-segmentation-based brain tumor segmentation for MRI data, followed by a hybrid method combining Vision Transformer (ViT) and Generative Adversarial Networks (GANs). This model aims to extract both global and localized features effectively while addressing challenges like data scarcity, high computational costs, and limited discrimination capability. Tested on BraTS 2020 and Masoud2021 datasets, it achieved high accuracy (97.65% and 98.99%) as well as sensitivity (97.7% and 96.83%). This hybrid model aims to enhance the efficiency and accuracy of brain tumor detection and segmentation, ultimately improving patient outcomes in clinical settings.

Tiwary et al. [112] developed a brain tumor segmentation method, which uses EfficientNet as an encoder to the traditional UNet architecture to enhance it. The UNet’s original bottleneck and skip connections were maintained to compress features and preserve spatial detail during upsampling. The model was evaluated on the Brain-Tumor.npy dataset containing 3064 contrast-enhanced T1-weighted MRI slices of three tumor types: meningioma, glioma, and pituitary tumor. Experimental results demonstrated strong performance, achieving an accuracy of 99.25% and a loss of 29.91%, showing that the proposed hybrid model outperforms or matches recent CNN and Transformer-based segmentation techniques. These hybrid techniques for brain tumor segmentation are summarized in Table 11.

Transformer-enhanced models, such as TransAttU-Net [108], which achieved a Dice coefficient of 98.87%, combine traditional U-Net structures with attention mechanisms to incorporate global contextual information, which is critical for accurately segmenting infiltrative brain tumors. Object detection networks, such as YOLO [110], sometimes perform poorly when segmenting tumors with irregular or diffuse borders, despite providing a high-speed performance with an accuracy of 93.6%. More recent approaches, such as GAN-ViT fusions [111], which reached an accuracy of 97.65%, integrate the generative capabilities of GANs with the hierarchical attention modeling of Vision Transformers to preserve fine structural details in both tumor and healthy tissues.

#### 3.3.3. Tumor Classification Using Hybrid Techniques

Srinivas et al. [113] presented a hybrid CNN-KNN model, which combined convolutional neural networks (CNNs) for feature extraction with a k-nearest neighbor (KNN) classifier for predicting tumor classes using MRI data. The model produced a classification accuracy of 96.25% when evaluated on the BraTS 2015 and 2017 datasets. Chelghoum et al. [114] presented a deep transfer learning method for classifying brain malignancies from contrast-enhanced MRI (CE–MRI) images of pituitary, glioma, and meningioma tumors using convolutional neural networks (CNNs). They adopted deep transfer learning by leveraging nine pretrained CNN architectures to improve classification accuracy, reduce training time, and avoid overfitting. Using Figshare dataset, this method showed a score of 98.71% for classification accuracy, which gives it the potential to significantly enhance clinical practices related to brain tumor diagnosis and management. Using deep feature extraction and machine learning classifiers, Kang et al. [115] presented a technique to classify tumors from brain MRIs. They then used a variety of pretrained convolutional neural networks (CNNs) and transfer learning to create features from MR images. For each classifier, the most important sets of deep features were selected and put together as an ensemble, which was then fed into other machine learning classifiers to predict the kinds of tumors. It showed classification accuracies of 92.37%, 96.13%, and 84.87%, respectively, on BT-small-2c, BT-large-2c, and BT-large-4c datasets.

In order to overcome the problem of limited medical imaging datasets, Ahmad et al. [116] proposed a framework for brain tumor classification that makes use of unsupervised deep generative neural networks. To generate the noise vector with additional information on the picture manifold, the methodology replaced the encoder–decoder network after a training phase with generative adversarial networks (GANs) and variational autoencoders (VAEs). A cascaded GAN, which can provide realistic MRI pictures of brain tumors and prevent mode collapse, was trained using this vector. This model reached an accuracy of 96.25% on 3064 CE-MR images dataset. In order to classify brain tumors from MRI images, Tummala et al. [117] presented a unique model based on a set of pretrained and refined Vision Transformer (ViT) models. MRI images (from the Figshare dataset) were downsized to 224 × 224 and 384 × 384 resolutions and then processed using the ViT models (B/16, B/32, L/16, and L/32), which were pretrained on ImageNet. The best individual accuracy was achieved by the L/32 model (98.2%), while the ensemble achieved 98.7%, proving the usefulness of ViT models for classifying brain tumors and their potential to support radiologists.

Soni et al. [118] presented a hybrid framework that integrated RFC (random forest classification) and CNNs (convolution neural networks) for the classification of brain tumors. The CNN mode automatically extracts features (non-hand-crafted feature extraction). Then, using a variety of classifiers, including Naïve Bayes (NB), k-nearest neighbor (KNN), support vector machine (SVM), random forest (RFC), and decision tree (DT), the extracted features were utilized to predict the kinds of tumors. After being tested on a dataset of 2870 MRI pictures, the suggested model produced an accuracy of 92.51% overall. To improve the accuracy of tumor classification in brain MRI images, Nassar et al. [119] created a model that integrates five pretrained convolutional neural network (CNN) models: GoogleNet, AlexNet, SqueezeNet, ShuffleNet, and NasNet-Mobile. Using 3064 T1-CE MRI dataset, this approach demonstrated a 99.31% classification accuracy. This model can lead to improved diagnostic accuracy, better patient management, and enhanced treatment outcomes for individuals with brain tumors. Dutta et al. [120] introduced a novel technique that combines pretrained CNN architectures with a global transformer module (GTM) to improve brain tumor classification from MRI images. They extracted feature maps using a backbone CNN, which were subsequently refined by the GTM using Generalized Self-Attention Blocks (GSBs), which capture long-range relationships via spatial and channel-wise attention processes. When validated on 3064 MRI images dataset, this model produced an accuracy of 97.11%.

Saha et al. [121] introduced BCM-VEMT as an improved classification framework that distinguishes different types of brain tumors through MRI images. They used a convolutional neural network (CNN) for automatic deep feature extraction from images followed by ML classifiers for tumor classification. To improve overall performance, the predictions from the ML classifiers are combined using a weighted average ensemble approach. The system classifies images into four categories: glioma, meningioma, pituitary, and normal. Trained on a dataset of 3787 MRI images, the BCM-VEMT model showed accuracy rates of 97.90%, 98.94%, 98.94%, and 98.00%, respectively, for the glioma, meningioma, pituitary, and normal classes, showcasing its effectiveness as a computer-aided diagnostic tool for brain cancer. These hybrid techniques for brain tumor classification are summarized in Table 12. 

CNN ensembles [119] outperform individual models with an accuracy of 99.31%, by utilizing a variety of feature representations from several networks, which improves generalization and resilience. However, enhanced performance comes at the expense of increasing computational complexity. The Global Token Mixer (GTM) represents transformer-based modules that achieve high accuracy rates of 97.11% through their ability to detect long-range relationships in MRI images for multi-focal malignancy identification. The diagnostic accuracy differences between glioma and meningioma cases persist due to the challenges introduced by varying textures within each category. The deep ensemble strategy BCM-VEMT [121] shows exceptional performance by reaching 97.9% accuracy while solving such variability issues through features specific to each class.

#### 3.3.4. Survival Prediction Using Hybrid Techniques 

Sun et al. [122] used a thorough approach that included deep learning techniques for glioma patient survival prediction and tumor segmentation. For tumor segmentation, they combined three different 3D CNN architectures—3D U-Net, DFKZ Net, and CA-CNN—using a majority voting approach to improve accuracy and robustness. A random forest regression model for survival prediction was then constructed using the selected features and clinical data. Using the BraTS 2018 dataset, the model’s prediction accuracy for patient survival was 61%. A completely deep-learning-based system was presented by Yogananda et al. [123] for tumor segmentation and patient survival prediction. Their approach uses three 3D Dense UNets for segmenting total tumor (WT), tumor core (TC), and enhancing tumor (ET) areas, respectively, employing different loss functions and data augmentation strategies to solve class imbalance while maintaining generalization. They used the linear regression and the Pyradiomics package to obtain imaging characteristics in survival prediction task. This methodology showed an accuracy of 55% on the BraTS 2019 dataset. In order to segment brain tumors from MRI images and predict patient survival, Amian et al. [124] used a multi-resolution 3D convolutional neural network (CNN). Their approach included two parallel CNN flows; one working at the original resolution for local features and the second at the lower resolution for global ones. For survival prediction, spatial features were extracted and passed to the model random forest regressor. On the BraTS 2019, their model showed Dice scores of 84%, 74%, and 71% for various tumor locations and survival prediction accuracies of 52% for validation and 49% for testing sets.

Nawaz et al. [125] created a model that employed machine learning for prognosis using MRI data and deep learning for tumor segmentation. They segmented brain tumors from MRI images using the VGG19-UNET model, which leverages the VGG19 convolutional neural network as the encoder for improved feature extraction. Then, random forest and Naïve Bayes classifiers were combined for patient survival prediction, yielding an accuracy of 62.7% on the BraTS 2020, enhancing tumor delineation, and aiding in personalized treatment planning. Rafi et al. [126] introduced a methodology for predicting overall survival (OS) time and segmenting brain tumor in MRI images. For brain tumor segmentation, they used a 3D multi-level dilated convolutional neural network (MLDCNN) based on a modified U-Net. Radiomic features based on texture, shape, and volume were recovered from the segmented tumor components in axial coronal and sagittal views of MRI slices in order to estimate the OS time, addressing the problem of loss of information when features are generated from a single image. These multi-view features were then fed into a group of three random forest regressor (RFR) models that learned to generalize predictions better reducing mean squared error. This approach showed an OS prediction accuracy of 48.3% on BraTS 2019. Huang et al. [127] created a new model to predict glioma patient survival using deep learning with clinical data. To segment three sub-regions of brain tumors in multi-modal MRI imaging, they developed the NLSE-VNet model, which combines the Non-Local and Squeeze-and-Excitation modules into the V-Net architecture. Together with clinical information such as tumor grade and patient age, they used the Pyradiomics toolbox to extract radiological features, CNN to caption deeper features, and a random forest regression (RFR) model to predict survival. The suggested technique demonstrated a 79% Dice score for the segmentation task and a Root Mean Square Error (RMSE) of 311.5 for survival prediction when tested on the BraTS 2019 and 2020 datasets.

Asthana et al. [128] presented a novel approach that integrated deep learning for tumor segmentation with a unique regression model for survival prediction. They employed a modified U-Net architecture for semantic segmentation of the brain tumors in MRI images and a novel regression model to forecast the survival rates of patients with brain tumors. On the BraTS 2018, 2019, and 2020 datasets, the suggested approach yielded prediction accuracies of 64.2%, 59.8%, and 60.5%, respectively. It enhances the diagnostic and prognostic capabilities in the management of brain tumors, which improve patient outcomes and streamline clinical processes. By blending radiomics features with clinical attributes, Zhou et al. [129] created a method for utilizing MRI data to predict the survival prospects of patients with medulloblastoma. They collected information from 217 subjects, employed a 3D-Unet model to perform MRI preprocessing and segmentation. Statistical approaches such as ICC, random survival forest, and LASSO regression method were implemented to choose features. The Cox proportional hazards model was used for developing a radiomics signature (Rad-score), which was integrated with clinical characteristics into an active nomogram that predicts the overall survival rate. This approach yielded better results with a C-index of 74.7%, which surpassed other models based on using only clinical features or radiomics.

Zaitoon et al. [130] developed a hybrid framework that combines tumor detection along with classification and segmentation alongside survival rate prediction through the BraTS dataset. They preprocessed data using Convolutional Normalized Mean Filter (CNMF) to improve image quality after data acquisition. The DBT-CNN model handles multi-class tumor classification, while the RU-Net2+ model performs precise tumor segmentation. A Cox regression model was used to analyze key features obtained through segmentation of the images. These features are then input into a logistic regression model to predict patient survival rates. The framework presented high survival prediction accuracy rates reaching 85.71% for long-term, 72.72% for medium-term, and 61.54% for short-term survivors, showing its potential to assist clinicians in treatment planning and decision making. These hybrid techniques for brain tumor survival prediction are summarized in Table 13.

Studies combining CNN-based imaging features with radiomic descriptors and clinical data have outperformed unimodal approaches by producing a 12% increase in C-index performance [127]. The models exploit rich additional data sources while their performance depends on the accuracy of manual segmented tumors, which introduces measurement inconsistencies. Traditional ensemble techniques, such the random forest and Naïve Bayes classifier combination [125], which achieves 62.7% accuracy, are more resilient to incomplete or noisy data. However, they frequently fail to predict short-term survival outcomes, highlighting the clinical unpredictability and molecular complexity of aggressive gliomas.

## 4. Conclusions

In brief, the combination of advanced imaging technologies and artificial intelligence has transformed brain tumor evaluation by enhancing precision significantly in the diagnosis as well as management plan. Automated and refined analysis of brain tumors using machine learning tools from MRI remains important, yet advances have taken place, for example in the region of standards such as re-defining/labeling measurements. Ultimately, this review demonstrated the value of using algorithms by applying them to different tumor analysis stages; from detection and prediction considering each stage highlighted how complicated an essential clinical evaluation can become. Despite the progress and advancements in this field, challenges such as large, annotated datasets required for training models and variability in imaging protocols still remain. Follow-up studies should work through these difficulties and find algorithms/strategies for an enhanced conciseness of brain tumor diagnosis and treatment. In particular, the development of novel hybrid models, such as the coupling of interpretable machine learning classifiers with lightweight deep learning architectures, might improve both efficiency and clinical transparency, exploring novel algorithmic combinations beyond standard frameworks like CNNs and transformers or incorporating domain-specific knowledge (e.g., radiomics or genomic data) into the learning process represents a promising direction for impactful contributions. Further cooperation between clinicians and data scientists will be crucial for the development of new techniques beneficial to patient outcomes influencing their chance of survival.

## Figures and Tables

**Table 1:** MRI sequences, Preston (1997).

MRI Sequence	Reception Time (TR)	TR Type	Time to Echo (TE)	TE Type
T1-weighted	500 ms	Short	14 ms	Short
T2-weighted	4000 ms	Long	90 ms	Long

**Table 2:** Machine-learning-related works for brain tumor detection.

References	Used Method	Datasets	Accuracy
[25]	feed-forward back-propagation neural network	239 MRI images	99%
[26]	K-means clustering	BraTS2015	94.07%
[27]	K-means clustering and SVM	Harvard, RIDER, and Local	97.1%
[28]	NSCT and ANFIS	BRATS 2015 Leaderboard and Challenge	95.9% and 96.4%
[29]	Naïve Bayes classification	50 MRI images	94%
[30]	Adaptive boosting and random forest models	Radiomic features from 167 patients	87.5%
[31]	Fuzzy c-means clustering, Extreme Learning Machine	Figshare and a combined dataset from Figshare, SARTAJ, and Br35H datasets	98.56%

**Table 3:** Machine-learning-related works for brain tumor segmentation.

References	Used Method	Datasets	Accuracy
[32]	Differential Evolution (DE), the OTSU thresholding, and the neural networks	images acquired from 56 patients	94.73%
[33]	Extreme Learning Machine and regressional ELM	BRATS 2012–2015 datasets	95.7%
[34]	K-means++ and Gaussian kernel-based FCM	100 images dataset	94.6%
[35]	K-means clustering	KICA and BraTS 2015	96%
[36]	ELM and a fast FCM clustering	TCGA-GBM and REMBRANDT	98.75%
[37]	K-means and FCM clustering	BrainWeb and BRATS 2015	96.5%
[38]	K-means and FCM clustering	Collected dataset from patients in ANBU hospital Madurai	-
[39]	Combined machine-learned features	BRATS 2017	-

**Table 4:** Machine-learning-related works for brain tumor classification.

References	Used Method	Datasets	Accuracy
[40]	KNN, RF, SVM, and LDA	REMBRANDT	90% for SVM method
[41]	k-nearest neighbor (KNN)	TCIA	89.5%
[42]	Bag of Features module	TCIA, XNAT and Oasis	97%
[43]	KSVM and SSD classifiers	BRATS 2018, 2019, and 2020	99.2%, 99.36%, and 99.15%
[44]	kernel-based support vector machine (K-SVM)	160 MRI images	98.75%
[45]	SVM with RBF and linear kernels	Dataset derived from internet	87.5%
[46]	Five ML models	1000 MRI images	98.51% for K-NN

**Table 5:** Machine-learning-related works for brain tumor survival prediction.

References	Used Method	Datasets	Accuracy
[47]	Support vector machine (SVM)	BraTS dataset	66.7%
[48]	Random forest regression model	163 cases from the BraTS17	63%
[49]	Stationary Wavelet Transform	BraTS 2018	69.5%, 67.1%, and 55.8%
[50]	random survival forest (RSF) model	TCGA and TCIA datasets	70.9%
[51]	KNN model	BraTS 2020	64.4%
[52]	Machine learning model	BraTS 2020	53.8% and 55.2%

**Table 6:** Deep-learning-related works for brain tumor detection.

References	Used Method	Datasets	Accuracy
[53]	Deep CNN (DCNN)	3394 MRI images	98.22%
[54]	CNN	3264 MR images	93.3%
[55]	Dense EfficientNet	3260 T1-weighted MRI	98.78%
[56]	Residual CNN	BraTS 2015	94.43%
[57]	CNN	Figshar, SARTAJ, and Br35H	95.44%
[58]	Optimized CNN	400-image dataset	90%
[59]	Faster R-CNN	233 patients’ dataset	77.60%
[14]	LeU-Net	253 MRI images	98% and 94%
[60]	U-Net CNN	3264 MRI images	98.67%
[61]	Deep learning (DL)	Images from 233 patients	98.95%
[62]	DL	10.000 brain MRI images	98.28%
[63]	YOLOv7	10.288 images	99.5%
[64]	Transfer learning and CNN	three MRI datasets	100%, 97% and 84.78%
[65]	multi-layer CNN	2.870 MRI images	91.3%
[66]	RViT	Figshare, SARTAJ, and Br35H datasets	98.6%
[67]	FT-ViT	CE-MRI	98.13%
[68]	VITALT	Four different datasets	99.08%, 98.97%, 98.82% and 99.15%

**Table 7:** Deep-learning-related works for brain tumor segmentation.

References	Used Method	Datasets	Accuracy
[69]	Dilated convolution and multi-task learning	BraTS 2018	85.87%
[70]	Cascade CNN (C-CNN)	BraTS 2018	-
[71]	optimized 3D U-Net-based architecture	BraTS 2020	-
[72]	Znet	TCGA-LGG	99.6%
[73]	3D U-Net model and a 3D CNN	Figshare	99.40% and 100%
[74]	3D U-Net based CNN	BraTS	98%
[75]	CNNs	1000 scans	91% and 89%
[76]	Swin-UNet-EPA	BraTS 2021	-
[77]	Generative Adversarial Network (GAN)	BraTS 2020	-

**Table 8:** Deep-learning-related works for brain tumor classification.

References	Used Method	Datasets	Accuracy
[78]	Deep transfer learning model	Figshare	98%
[79]	CNN and transfer learning	1572 T1w MRI images	-
[80]	Deep transfer learning	Figshare	99.67% and 95.87%
[81]	CNNs	BraTS2018	97.3% and 96.5%
[82]	ConvNet	3064 MRI images	98.04%
[83]	25-layer CNN	3064 MRI images	86.23% and 81.6%
[84]	DL (ResNet and U-Net)	BRATS 2020	97. 8% and 96.9%
[85]	NeuroNet19	7023 MRI images	99.3%
[86]	CNN and a soft attention mechanism	Figshare	-
[87]	Ensemble deep learning and transfer learning	BraTS	98.3%, 98.6%, and 98.6%
[88]	Attention-Gated Recurrent Units (A-GRU)	BTD dataset	99.32%
[89]	Deep CNN	7000 MRI images	94–97%

**Table 9:** Deep-learning-related works for brain tumor survival prediction.

References	Used Method	Datasets	Accuracy
[90]	3D deep CNN	BraTS dataset	74%
[91]	DL (InceptionResNetV2)	124 patients’ data	86%
[92]	dCNN	500 paired images	66.7%, 67.3%
[93]	deep learning encoder–decoder ConvNet	BraTS 2018	54%
[94]	Attention-CNN based 3D U-Net	BraTS 2019	48.3% and 38.3%

**Table 10:** Hybrid techniques in related works for brain tumor detection.

References	Used Method	Datasets	Accuracy
[95]	Hybrid CNN	BraTS 2013 dataset	-
[96]	2D CNN, convolutional auto-encoder network, and KNN	3264 MRI images	96.47%, 95.63%, and 86%
[97]	Ensemble Deep Neural Support Vector Machine (EDN-SVM)	255 T1-mode MRI pictures	97.93%
[98]	YOLOv5, K-means+, SPPF+, and Bi-FPN	3064 MRI images	-
[99]	CNN-based transfer learning classifiers and ViT models	7023 MRI images	-
[100]	Faster R-CNN with SVM classifier	BraTS 2015	98.81%
[101]	CNNs + GANs	3064 CE-MR images	98.85%
[102]	Deep Transfer Learning	7023 MRI images	99.75%

**Table 11:** Hybrid techniques in related works for brain tumor segmentation.

References	Used Method	Datasets	Accuracy
[103]	CNN and Multi-Class SVM (M-SVM)	40 MRI images	84%
[104]	Hybrid SegNet, U-net and ResNet18 architectures	BraTS dataset	97.62%, 98.19%, 98.08%, 98.24%, 98.85%, and 99.11%
[105]	Five modified Unet architectures	BraTS-2019	-
[106]	Hybrid deep convolutional neural network (DCNN)	253 MR images	99.7%
[107]	ResNet-SVM	372 MRI images	89.36%
[108]	Transformer-based attention mechanism with a U-net model	BraTS 2019 and 2020 datasets	98.47%
[109]	UNet architecture and transformer-based modules	BraTS datasets	-
[110]	YOLOv5 and YOLOv7	Figshare dataset	-
[111]	ViT and GANs	BraTS 2020 and Masoud2021	97.65% and 98.99%
[112]	Traditional UNet + EfficientNet	The Brain-Tumor.npy dataset	99.25%

**Table 12:** Hybrid techniques in related work for brain tumor classification.

References	Used Method	Datasets	Accuracy
[113]	CNNs and KNN	BraTS2015 and 2017	96.25%
[114]	Deep transfer learning with convolutional neural networks (CNNs)	Figshare	98.71%
[115]	Deep feature extraction and machine learning	BT-small-2c, BT-large-2c, and BT-large-4c	92.37%, 96.13%, and 84.87%
[116]	VAEs and GANs	3064 CE-MR images	96.25%
[117]	Ensemble of pretrained and refined ViT models	Figshare	98.7%
[118]	CNN and RFC	2870 MRI images	92.51%
[119]	five pretrained CNN models	3064 T1-CE MRI images	99.31%
[120]	Pretrained CNN architecture and a global transformer module (GTM)	3064 MRI images	97.11%
[121]	CNNs + Machine learning	3787 MRI images	97.90%, 98.94%, 98.94%, and 98.00%

**Table 13:** Hybrid techniques in related works for brain tumor survival prediction.

References	Used Method	Datasets	Accuracy
[122]	DL techniques and random forest regression model	BraTS 2018	61%
[123]	3D Dense UNets and linear regression	BraTS 2019	55%
[124]	multi-resolution 3D CNN and Random Forest regressor	BraTS 2019	52% and 49%
[125]	DL and ML techniques	BraTS 2020	62.7%
[126]	CNNs and RFR	BraTS 2019	48.3%
[127]	DL models with RFR	BraTS 2019 and BraTS 2020	-
[128]	Modified U-Net with a novel regression model	BraTS 2018, 2019, and 2020	64.2%, 59.8% and 60.5%
[129]	3D-Unet with ML methods	Data from 217 medulloblastoma (MB) patients	-
[130]	RU-Net2+	BraTS	85.71%, 72.72%, and 61.54%
